# Organic porous heterogeneous composite with antagonistic catalytic sites as a cascade catalyst for continuous flow reaction†

**DOI:** 10.1039/d3sc03525e

**Published:** 2023-09-07

**Authors:** Sumanta Let, Gourab K. Dam, Sahel Fajal, Sujit K. Ghosh

**Affiliations:** a Department of Chemistry, Indian Institute of Science Education and Research Dr Homi Bhabha Road, Pashan Pune 411008 India sghosh@iiserpune.ac.in +91 20 2590 8076; b Centre for Water Research, Indian Institute of Science Education and Research Dr Homi Bhabha Road, Pashan Pune 411008 India

## Abstract

One-pot cascade catalytic reactions easily allow the circumvention of pitfalls of traditional catalytic reactions, such as multi-step syntheses, longer duration, waste generation, and high operational cost. Despite advances in this area, the facile assimilation of chemically antagonistic bifunctional sites in close proximity inside a well-defined scaffold *via* a process of rational structural design still remains a challenge. Herein, we report the successful fusion of incompatible acid–base active sites in an ionic porous organic polymer (iPOP), 120-MI@OH, *via* a simple ion-exchange strategy. The fabricated polymer catalyst, 120-MI@OH, performed exceedingly well as a cascade acid–base catalyst in a deacetylation-Knoevenagel condensation reaction under mild and eco-friendly continuous flow conditions. In addition, the abundance of spatially isolated distinct acidic (imidazolium cations) and basic (hydroxide anions) catalytic sites give 120-MI@OH its excellent solid acid and base catalytic properties. To demonstrate the practical relevance of 120-MI@OH, stable millimeter-sized spherical composite polymer bead microstructures were synthesized and utilized in one-pot cascade catalysis under continuous flow, thus illustrating promising catalytic activity. Additionally, the heterogeneous polymer catalyst displayed good recyclability, scalability, as well as ease of fabrication. The superior catalytic activity of 120-MI@OH can be rationalized by its unique structure that reconciles close proximity of antagonistic catalytic sites that are sufficiently isolated in space.

## Abstract 2

Ionic porous polymer featuring antagonistic catalytic sites as a cascade catalyst for continuous flow reaction.

## Graphical abstract

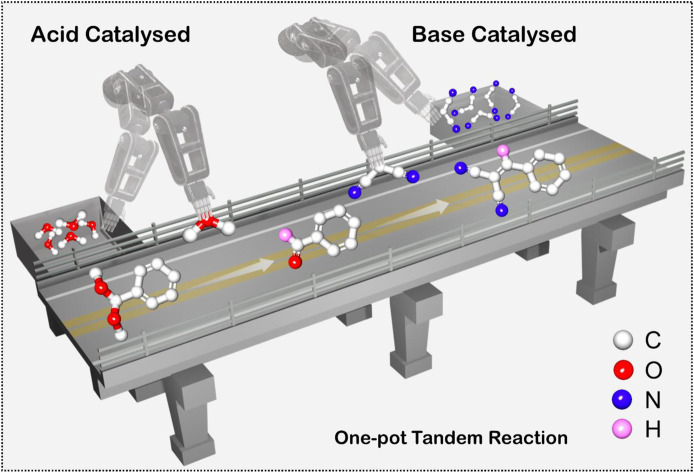

## Introduction

Catalytic cascade reactions (A → B → C…) have emerged as an elegant and efficacious technique to achieve one-pot multi-step organic transformations. Additionally, cascade reactions provide an advantage with regard to reduced waste generation and result in overall shortened reactions and decreased operational costs.^1–4^ Having said that, progress in one-pot tandem catalysis has been thwarted by difficulties in mainly two aspects. Firstly, the rational design of catalysts compatible with one-pot tandem protocols is arduous, particularly for those reactions comprising successive organic transformations. These transformations demand synergistic catalytically active sites that require antagonistic, or conflicting, properties without mutual intrusion, *i.e.*, acidic sites in step 1 (A → B) and basic sites in step 2 (B → C), such as a deacetalization-Henry reaction, deacetalization-Aldol reaction, deacetalization-Knoevenagel condensation reaction *etc.*^5–8^ Secondly, tethering antagonistic catalytic sites necessitates a rigid heterogeneous nanoarchitecture. For example, core–shell type or yolk–shell nanomaterials are used but often prove suboptimal due to limited active site accessibility.^9–13^ However, nature has found its unique way to mitigate this problem *via* compartmentalization, *i.e.*, spatial isolation of catalysts in the nano-terrain of organelles in living cells. This spatial partitioning inhibits cross-reactions in catalysts (as well as substrates), allowing them to engage in reactions in a specific order, leading to the desired product of the catalytic reaction.^14^ Taking inspiration from this catalyst site-isolation that exists in nature, researchers around the globe have tried to revamp catalyst design by separately storing catalysts in distinct repositories^15^ such as linear polymers,^16^ polymeric nanostructures (*e.g.* micelles,^17^ polymersomes,^18,19^ hydrogels,^20^ bottle-brushes,^21^ Pickering emulsions,^22,23^ graphene oxide,^24^ silica particles yolk–shell,^25^ double shell,^26^ mesoporous silica).^27,28^ One initial report combines two star polymers containing both acid and base catalysts as an elegant approach to drive the one-pot cascade deacetalization-Henry reaction in DMF.^29^ On the other hand, in an attempt to diminish the diffusion path of the reactants from one catalytic centre to the adjacent one and thus, likely lower the reaction time, more research attention was directed to combining dual catalysts in the same material in only a few nanometers of interspace.^30–32^

**Scheme 1:**
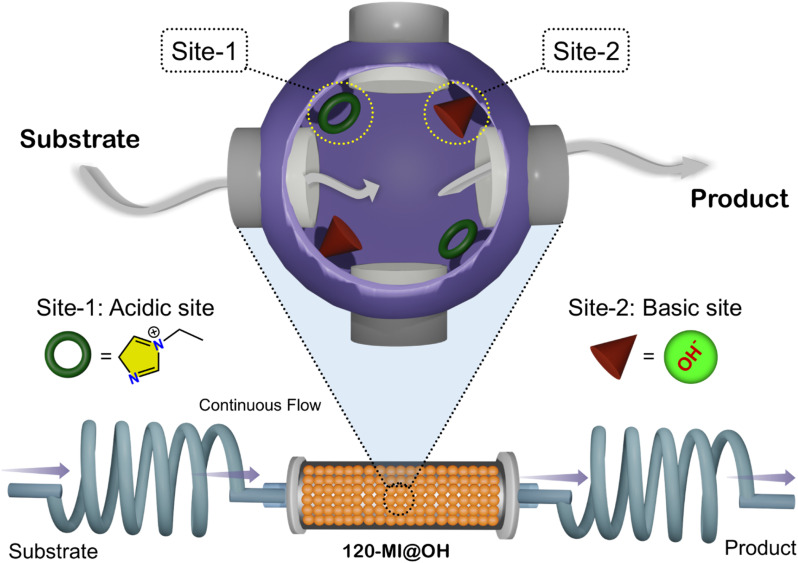
Schematic illustration of continuous flow one-pot antagonistic cascade catalysis using 120-MI@OH.

Although some reports have successfully combined antagonistic acid–base catalytic sites in a single material for cascade one-pot catalysis, there still remain pitfalls toward practical application. For example, complicated and tedious synthetic routes that demand protection and deprotection methodologies to avoid acid-base neutralization as well as the use of pore-forming agents to render easy mass transfer of reactants are still required.^33^ Other catalyst supports, *e.g.*, metal organic frameworks (MOFs) and graphene oxide, are difficult to use for on-demand post-synthesis functionalization owing to their bulk and rigidity, while for silica there is a lack of suitable silane precursors. Additionally, mesoporous silica is unstable in strong alkaline media and most MOFs decompose in acidic conditions.^34^ Hence, it is imperative to explore and develop potent heterogeneous bifunctional catalysts that have efficient activity and are sufficiently robust whilst being simple to synthesize and post-structurally functionalize to harness cooperative catalysis (Scheme 1).

Porous organic polymers (POPs) have emerged as an intriguing class of amorphous polymers that bear exceptional physiochemical stability and a large surface area. Additionally, POPs have diverse chemical compositions, desirable functionalities, and are easy to engineer post-synthesis and maintain control over pore size and volume. Consequently, they have established their utility in varied applications such as gas storage and separation,^35^ environmental remediation,^36,37^ and heterogeneous catalysis^38,39^*etc.* In heterogeneous catalysis, POPs feature advantages such as: (1) diverse functional groups can be integrated into the POP skeleton through chemical reactions;^40–42^ (2) the nanoscale confinement present in POPs gives them enhanced activity that is reflected in yield and selectivity of the product;^43^ and (3) POPs are generally insoluble, stable and easily recovered.^44^ Hyper-cross-linked microporous polymers (HCPs) have drawn great research attention owing to their unique properties, *i.e.*, high stability, convenience with which their functionality is tailored, facile and cost-effective synthesis, large permanent porosity, *etc.*^45,46^ These excellent properties have made them a fitting choice as a support matrix for crafting potent heterogeneous catalysts that exhibit enhanced performance and recyclability.^47,48^ Synthesizing fine chemicals in continuous flow has attracted much attention lately.^49^ To this end, continuous flow cascade catalysis represents a step-change in the methods available for the stepwise synthesis of fine chemicals. Continuous flow cascade catalysis encompasses both the convenience of a cascade reaction and continuous flow methodology combined with better reaction efficiency and lower energy utilization whilst removing the need for separation of the reaction intermediates and resulting in a beneficial shift in the reaction equilibrium.^50–53^ In the current work, with the aim of achieving the above mentioned fundamental goal we report the straightforward fabrication of an efficient heterogeneous polymer catalyst, 120-MI@OH, for the successful execution of a one-pot cascade deacetalization-Knoevenagel condensation in a continuous flow manner under mild conditions. This particular cascade reaction serves its usefulness in preparing coumarins and related derivatives that are crucial intermediates for the synthesis of pharmaceuticals, perfumes, cosmetics *etc.*^54^ Satisfyingly, 120-MI@OH displayed excellent performance in cascade catalysis under both batch and flow conditions. Additionally, owing to its structure that contains site-isolated antagonistic sites, the catalyst polymer demonstrated outstanding activity for both the cyanosilylation (acid catalyzed) and Knoevenagel condensation (base catalyzed) reactions.

## Results and discussion

### Catalyst synthesis and structural characterization

**Fig. 1:**
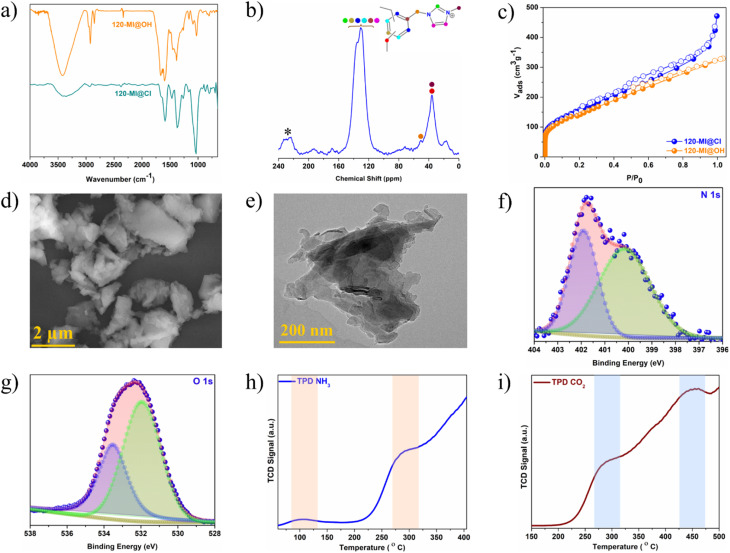
(a) FT-IR spectra of 120-MI@Cl (cyan) and 120-MI@OH (orange). (b) Solid-state ^13^C CP/MAS NMR spectra of polymer 120-MI@Cl. (c) N_2_ sorption curves of 120-MI@Cl (blue) and 120-MI@OH (orange). (d) FESEM image of 120-MI@OH. (e) TEM image of 120-MI@OH. (f) N 1s XPS spectrum of 120-MI@OH and (g) O 1s XPS spectrum of 120-MI@OH. (h) NH_3_ TPD profile and (i) CO_2_ TPD profile of 120-MI@OH.

Schemes S1–S3† illustrate the synthesis of the polymer catalyst 120-MI@OH *via* strategic post-synthesis functionalization. Precursor polymer 120-Cl was synthesized by our previously published method.^55^ Following this, imidazolium ionic liquid moieties were incorporated through a nucleophilic substitution reaction at the electrophilic chloromethyl centres using *N*-methylimidazole to produce 120-MI@Cl. In the second step, a straightforward ion-exchange reaction was used to introduce hydroxyl anions (OH^−^) throughout the polymer network by treating 120-MI@Cl with NaOH solution. These stepwise structural modifications resulted in immobilized imidazolium cations and hydroxyl anions, forming Lewis acid–base pair antagonistic catalytic sites in the 120-MI@OH structure. After their successful synthesis, the polymers were comprehensively characterized and their structures verified using various techniques and analyses, such as Fourier transform infra-red (FTIR) spectroscopy, solid-state NMR spectroscopy, low temperature nitrogen sorption isotherms (N_2_ adsorption), thermogravimetric analysis (TGA), field emission scanning electron microscopy (FESEM), transmission electron microscopy (TEM), and X-ray photoelectron spectroscopy (XPS) *etc.* The recorded FT-IR spectrum of 120-MI@Cl verified the successful integration of imidazolium cations inside the polymer network. Peaks arising at *ca.* 1662 cm^−1^ and 2922 cm^−1^ are ascribed to the olefin C=C bonds from the aromatic rings whilst two peaks at around 1163 cm^−1^ and 1598 cm^−1^ were assigned to the C–N and C=N bonds from the imidazole moiety, respectively, confirming the quaternization reaction had proceeded successfully. The appearance of an additional broad peak at *ca.* 3420 cm^−1^ in 120-MI@OH can be attributed to the stretching frequency of the hydroxyl group, indicating the successful fabrication of the desired polymer catalyst (Fig. 1a and S1†). It should be mentioned that both 120-MI@Cl and 120-MI@OH displayed distinct stretching frequencies at *ca.* 1460 cm^−1^ and 2920 cm^−1^ that correspond to the C–H bending and stretching vibrations, respectively, owing to the presence of cross-linking –CH_2_ linkages throughout the polymer matrix. The structural composition was probed *via* solid-state ^13^C cross-polarization magic-angle spinning (CP/MAS) NMR spectroscopy. Presence of three main broad peaks at 36 ppm, 129 ppm and 135 ppm was observed for 120-MI@Cl, corresponding to the presence of methylene carbon from the external crosslinker, the C4/C5-carbons of the imidazole ring and the non-substituted aromatic carbons, and the C2-carbon of the imidazolium ring along with the substituted aromatic carbons, respectively (Fig. 1b and S2†).^56,57^ Additionally, a peak at 51 ppm was assigned to the adjacent carbon atom attached to the N atom of the imidazole ring and the resonance peak at 16 ppm was attributed to the methyl carbon of the imidazole moiety, further validating the success of the post-synthesis modification. A porosity analysis was carried out by performing N_2_ sorption measurements at 77 K. Both 120-MI@Cl and 120-MI@OH displayed swift nitrogen uptake at very low pressure (*P*/*P*_0_ < 0.03) accompanied by a continuous increase in uptake at high relative pressures, thus indicating hierarchical porosity featuring both micropores as well as mesopores (Fig. 1c and S7†).^58^ A similar pattern of initial weight loss under 100 °C was seen for all the pristine polymers, after which a retention of greater than 70 wt% up to ∼400 °C demonstrated their thermal robustness (Fig. S3–S6†). While the first weight loss was assigned to the removal of trapped solvent molecules, the gradual loss after 400 °C accounts for the decomposition of the organic frameworks. The surface morphology of the polymers was evaluated *via* FESEM and TEM analysis. The FESEM images revealed amorphous dense particles of irregular shape in the case of the catalyst polymer 120-MI@OH (Fig. 1d). Furthermore, no significant change in the surface morphology was noticed in the modified polymers compared to the pristine polymer (Fig. S8†). The homogeneous presence of all the compositional elements in all the polymers was confirmed *via* EDX analysis coupled with elemental mapping, which additionally confirms the success of the post-synthetic structural modifications (Fig. S9–S11†). TEM images of 120-MI@OH showed aggregated uneven-sized nanoparticles similar to those seen in the FESEM images (Fig. 1e and S12†). The modified polymer 120-MI@OH was further structurally characterized in detail using X-ray photoelectron spectroscopy (XPS) analysis. The survey-scan spectrum is shown in Fig. S13,† demonstrating three signals approximately at 285.2, 401.1, 533.1 eV that correspond to the C 1s, N 1s and O 1s elements in 120-MI@OH. After a detailed analysis, the high-resolution N 1s spectrum was deconvoluted to two distinct peaks, implying the abundance of two types of N of the imidazolium unit (Fig. 1f and S15†). Specifically, the peak at 401.8 eV corresponds to the cationic N^+^ atoms of the imidazolinium fraction while the other peak at 400.1 eV was ascribed to non-ionic N.^59–61^ The C 1s XPS spectrum was fitted into two different peaks at 285.8 eV and 284.6 eV, which were assigned to signals from the C–N of the imidazolium rings as well as the methylene and phenyl carbons of 120-MI@OH (Fig. S14†). These observations further confirm the integration of the imidazolium units in the structure. Overall, these observations further supported that the structural modification had enabled successful catalyst fabrication (Fig. 1f, g and S13–S16†). The existence of Lewis acidic and basic sites was further investigated by performing temperature-programmed desorption (TPD) spectrometry. The NH_3_-TPD profile of 120-MI@OH displayed two definite peaks at 104 °C and 300 °C attributable to both moderate and strong Lewis acidic sites, respectively (Fig. 1h). Similarly, the CO_2_-TPD analysis exhibited peaks at 276 °C and 455 °C corresponding to moderate as well as strong Lewis basicity (Fig. 1i). These experimental observations confirm the presence of antagonistic reaction sites in the fabricated catalyst.

### Base catalyzed Knoevenagel condensation under mild-conditions

The Knoevenagel condensation of carbonyls and active methylene compounds is a typical C=C bond forming reaction.^62^ Over the years, it has found utilization as a powerful and widely used reaction for the synthesis of fine chemicals, cosmetics, agrochemicals, pharmaceuticals, and biologically relevant heterocyclic compounds, *etc.*^63,64^ Generally, the Knoevenagel condensation serves the purpose of a reference reaction for heterogeneous basic catalysts.^65^ Motivated by the presence of Lewis basic sites in 120-MI@OH, we investigated the potential of 120-MI@OH as a catalyst in the Knoevenagel reaction by employing benzaldehyde and malononitrile as model substrates. Initially, a set of control experiments were performed to arrive at the optimal reaction conditions. As seen in Table S1,† when 0.8 mmol of benzaldehyde was allowed to react with 0.8 mmol of malononitrile in the presence of 120-MI@OH (5 mg), benzylidenemalononitrile was isolated in an excellent yield of up to 99% at room temperature (RT). It is also worth mentioning that amongst the tested solvents (H_2_O, CH_2_Cl_2_ and MeOH), H_2_O produced the best result (Table S1,† entries 1, 2, and 4) in only 30 minutes. Also, increasing the reaction time or catalyst amount did not have any significant effect on the yield (Table S1,† entry 3). 120-MI@Cl was found to catalyze the reaction but in a suboptimal yield (Table S1† entry 5).

Also, no significant conversion to the desired product was observed in the absence of 120-MI@OH, showing the important role of the catalyst (Table S1,† entry 6). The heterogeneous nature of the catalyst was confirmed by carrying out a control reaction with the supernatant of an H_2_O suspension of 120-MI@OH. This reaction resulted in almost no conversion and also indicated no leaching of catalytically active components of the catalyst (Table S1,† entry 7). These results clearly suggest the potency of 120-MI@OH as an efficient heterogeneous catalyst for the Knoevenagel condensation reaction. After arriving at the optimal reaction conditions, we evaluated the ability of 120-MI@OH to catalyze the conversion of a series of aromatic carbonyls to establish its universality. Table S2† illustrates the exceptional substrate scope and functionality tolerated by the polymer catalyst when tested with various aldehydes. However, a slightly decreased yield was observed for substrates with electron-donating groups such as CH_3_, –N(CH_3_)_2_*etc.* (Table S2,† entries 1g, 1h). On the other hand, substrates with electron-withdrawing groups produced excellent yields (up to >99%). When heterocyclic compounds were subjected to the reaction conditions (Table S2,† entry 1j), a high yield of 94% was achieved, which has profound importance in terms of synthesizing bioactive compounds of biological relevance. Additionally, we observed similar catalytic activity with ethyl cyanoacetate, suggesting the versatile nature of our polymer catalyst. ^1^H NMR spectra of all the desired compounds corroborated their purity (details in the ESI†).

### Acid catalyzed cyanosilylation under mild-conditions

Cyanohydrins have emerged as critical and versatile intermediates in biology and industry on account of being precursors for β-hydroxy amino alcohols, α-hydroxy carboxylic acids, α-hydroxy aldehydes and various fine chemicals.^66^ To synthesize cyanohydrins involves a nucleophilic addition with carbonyl compounds, and trimethylsilyl cyanide (TMSCN) is employed, replacing toxic chemicals such as HCN, NaCN or KCN, to generate a C–C bond *via* a greener approach.^67^ Organic–inorganic salts,^68^ nucleophilic catalysts^69^ or Lewis acids^70,71^ have been utilized to serve as electrophilic catalysts in order to activate various carbonyl compounds for efficient cyanosilylation reactions. Considering the Lewis acidity shown by 120-MI@OH, we evaluated its potential in catalytic cyanosilylation reactions. Reaction condition optimization was carried out by taking benzaldehyde (1 mmol), Me_3_SiCN (2.0 mmol), and the 120-MI@OH catalyst (5 mg) at room temperature (RT) without any solvent. After 0.5 h of reaction time, we observed a >99% yield of the cyanohydrin trimethylsilyl ether product. This performance can be ascribed to the excellent potential of 120-MI@OH to act as a Lewis acid solid catalyst. Motivated by this observation, we extended the substrate scope to various carbonyl compounds bearing diverse functionalities. It was observed that electron-withdrawing functionalities on the benzaldehyde moiety, such as –NO_2_, –CF_3_, –CN, and –Br (Table S3,† entries 2b, 2c, 2e, and 2g), did not have any significant impact irrespective of their position (*ortho*, *meta*) and produced near quantitative yields (Table S3,† entries 2c, 2l, and 2m). Likewise, electron-donating groups resulted in high to exceptional yields, validating the ability of 120-MI@OH as an efficacious heterogeneous catalyst for the cyanosilylation reaction under mild conditions (Table S3,† entries 2d, 2f, and 2h). Further, all the desired products formed were confirmed using ^1^H NMR spectroscopy (details in the ESI†).

### One-pot cascade deacetalization-Knoevenagel condensation

**Fig. 2:**
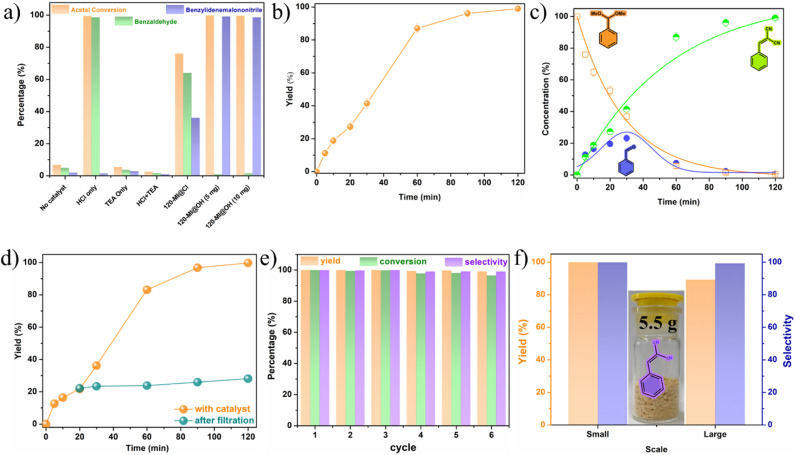
(a) Control experiments for the one-pot deacetalization-Knoevenagel condensation using different catalysts. (b) Kinetic profile of the cascade catalysis using 120-MI@OH. (c) Time-dependent catalytic performance of 120-MI@OH monitored over time using NMR. (d) Hot filtration test for one-pot deacetalization-Knoevenagel condensation. (e) Catalytic recycling test of 120-MI@OH for cascade reaction and (f) reaction yield comparison of small-scale and scale-up syntheses with a photograph showing the isolated pure product.

After successfully verifying the potency of 120-MI@OH as a bifunctional acid–base heterogeneous catalyst, we went ahead to investigate its performance in the one-pot cascade deacetalization-Knoevenagel condensation. This type of C–C coupling reaction is critical in fine chemicals synthesis.^72,73^ Benzaldehyde dimethyl acetal (0.5 mmol) and malononitrile (0.5 mmol) in 5 mL of H_2_O were selected as reactants for the benchmark reaction while the catalyst loading of 120-MI@OH (5 mg) was kept fixed. Typically, the first step of this cascade catalysis involves a deacetalization reaction to yield benzaldehyde from benzaldehyde dimethyl acetal and is promoted by the acidic catalyst sites. The second step consists of a Knoevenagel condensation with malononitrile, which is catalyzed by the basic sites to generate the unsaturated product, a crucial intermediate for the synthesis of pharmaceuticals, natural products, *etc.* After careful optimization of the ideal reaction conditions, we found that 120-MI@OH is remarkably active in the cascade catalysis and the desired benzylidenemalononitrile product was obtained with a 99% yield within just 2 h at 60 °C under aerobic conditions (Table S4†). Additionally, several control experiments were conducted to disseminate the reaction process (Fig. 2a). As seen in Fig. 2a, without addition of 120-MI@OH no desired product formation was observed, indicating the necessary role of the polymer catalyst. Subsequently, increasing the catalyst amount to 10 mg did not have a significant impact and the best activity was observed when using 5 mg of 120-MI@OH. The use of HCl (0.1 mmol) resulted in a smooth deacetalization reaction but the Knoevenagel condensation did not proceed to give the ultimate product. In contrast, using triethylamine (TEA, 0.1 mmol) led to a trace conversion of the acetal, showing the need for the acidic catalytic sites. However, a mixture of HCl and TEA produced almost no product owing to their neutralization reaction leading to the destruction of the antagonistic acid–base sites. ^1^H NMR was utilized to determine the product yield and conversion in all cases. These control reactions further confirm that spatially separated acidic and basic sites within the polymer catalyst is imperative in order to accomplish high performance cooperative catalysis. Additionally, 120-MI@Cl exhibited lower activity in comparison to 120-MI@OH, which may be attributed to its weaker basicity compared to 120-MI@OH.^74^

**Fig. 3:**
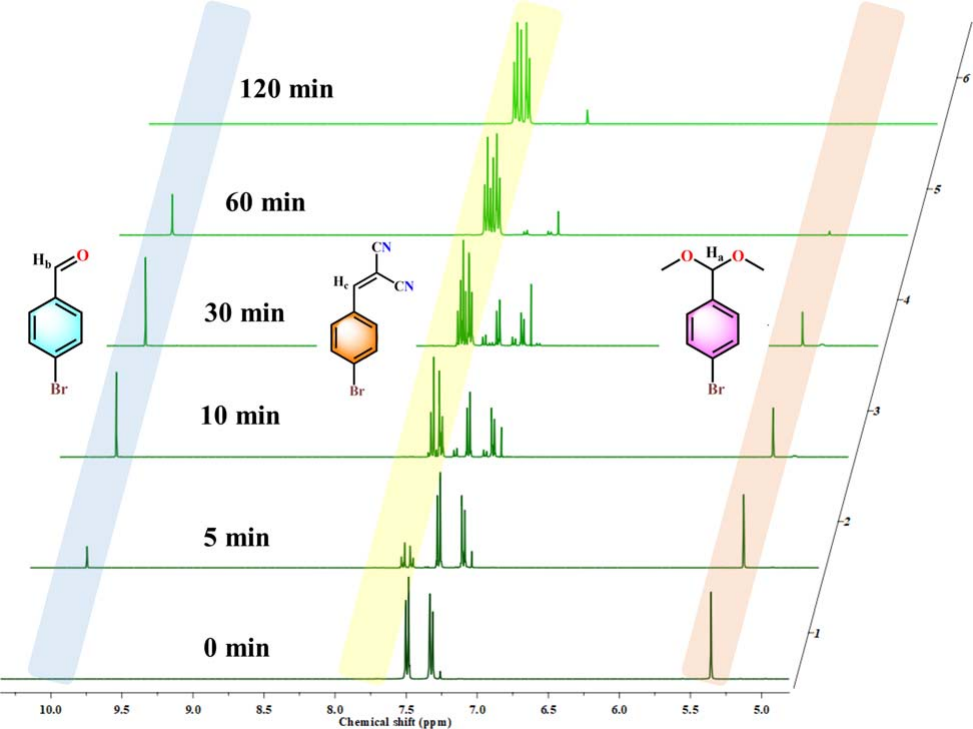
Partial ^1^H NMR spectra (CDCl_3_) of the cascade reaction at different time points (0 min, 5 min, 10 min, 30 min, 60 min and 120 min).

We also probed the kinetics of the reaction and determined the completion of the cascade catalysis. From Fig. 2b, a continuous incremental trend in the yield was seen over the course of time with >99% product yield after only 2 h. Each kinetic data point was duplicated to get accurate results. Also, the heterogeneity of 120-MI@OH was tested by removing the polymer catalyst after 20 min *via* filtration. The reaction was further allowed to proceed with the supernatant liquid but no significant increase in % yield of the product was observed, indicating that the reaction occurs in a heterogeneous manner (Fig. 2d). To delve deeper into the cascade reaction, we also monitored the time-dependent conversion of the reaction *via* plotting the consumption of benzaldehyde dimethyl acetal and the production of intermediate benzaldehyde, all the way to the final product benzylidenemalononitrile (Fig. 2c). This was carried out by taking out aliquots at predetermined time intervals from the reaction mixture while the conversion was monitored by ^1^H NMR spectroscopy. As illustrated in Fig. 2c, in accordance with the kinetics results, an exponential decrease in the starting material from its initial value is observed (orange trace). Within the first 20 min, ∼50% of the benzaldehyde dimethyl acetal was consumed, a figure which went up to ∼95% after 60 min on account of the deacetalization reaction facilitated by the imidazolium moiety. Accordingly, the amount of benzaldehyde intermediate (purple trace) was found to increase over the same duration of time (30 min). However, we observed an exponential decrement of the intermediate after 30 min owing to its conversion to the final product. Additionally, the amount of benzylidenemalononitrile showed a steady increase over the course of the reaction until it reached a plateau when the starting material and intermediate approached depletion. The sequential nature of the cascade catalysis was also established by performing a time-dependent *in situ*^1^H NMR titration study. A swift diminishing of the (30 min) peak corresponding to the phenylic proton of the benzaldehyde dimethyl acetal indicated its hydrolysis, whilst the appearance of the intermediate aldehyde proton finally leads to the desired final product (Fig. 3). Pertaining to sustainability, the reusability of a catalyst is not only critical but is a necessity. After performing each cycle of the cascade catalysis, the catalyst could be easily recovered *via* centrifugation, following which it was washed with water and methanol before drying at 80 °C for 6 h. 120-MI@OH demonstrated exceptional recyclability performance, with >98% of the final product obtained after 6 catalytic cycles, along with excellent conversion and selectivity (>99%) (Fig. 2e, S27 and S28†). Scalability is another crucial aspect of a catalyst and is highly sought after. We tested the ability of our catalyst at large scale (80 times greater) and we were able to isolate 5.5 g of the product after 2 h of the reaction with an admirable yield (89%), thus underlining its excellent scalability (Fig. 2f). These results clearly highlight the superior nature of 120-MI@OH in terms of robustness, sustainability, and remarkable catalytic activity. The post-catalysis stability of 120-MI@OH was assessed by employing FESEM and FT-IR analysis to validate that no significant changes in either the surface morphology or stretching frequencies had occurred compared to the parent polymer catalyst (Fig. S17 and S18†).

### Continuous flow cascade catalysis

**Fig. 4:**
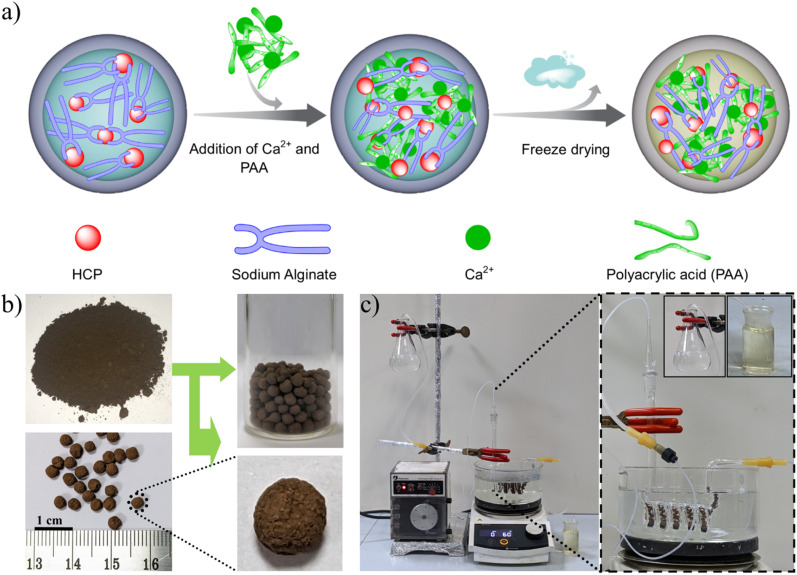
(a) Schematic illustration of PAA–Ca^2+^–alginate bead formation from 120-MI@OH powder. (b) Photographs of 120-MI@OH powder and beads respectively. (c) Photograph of the continuous-flow reactor for the synthesis of benzylidenemalononitrile at a gram scale.

Inspired by its satisfying catalytic performance, we tested the feasibility of performing the one-pot deacetalization-Knoevenagel condensation under continuous flow conditions. In this context, structuring of the polymer catalyst was done by converting 120-MI@OH powder into millimeter-sized composite beads using a double-cross-linking strategy utilizing poly(acrylic acid) (PAA), Ca^2+^ ions and sodium alginate with H_2_O as the solvent (Fig. 4a and b).^75^ After their synthesis, the composite beads were characterized to verify the successful integration of 120-MI@OH (Fig. S19†). Additionally, FESEM was employed to probe the surface morphology while SEM-EDX mapping illustrated a homogeneous distribution of Ca throughout the beads along with compositional elements of 120-MI@OH in the structured composite beads (Fig. S20 and S21†). 200 mg of polymer beads were packed in a custom made fixed microreactor to carry out the continuous flow catalysis. Typically, a benzaldehyde dimethyl acetal solution was continuously pumped through the inlet of the microreactor while the desired final product was collected from the reactor outlet. Under the optimized reaction conditions, an ethanolic solution (40 mL) of benzaldehyde dimethyl acetal (1.82 mL), malononitrile (792.8 mg) and 5 mL of H_2_O was allowed to flow at a flow rate of 0.4 mL min^−1^ to accomplish continuous conversion (Fig. 4c). To our delight, we obtained a 96% yield of the final product benzylidenemalononitrile on a gram scale (1.77 g). Additionally, a steady yield (>95%) of the desired product was obtained after performing 5 continuous flow catalytic cycles which demonstrates the excellent performance of 120-MI@OH in flow catalysis (Fig. S29 and S30†). The structural robustness of 120-MI@OH after performing the continuous flow catalysis was also verified through a combination of various techniques such as FTIR, TGA, FESEM, TEM and STEM-EDS mapping, giving a profile that shows the stability of the polymer catalyst (Fig. S22–S26†). Overall, the activity shown by 120-MI@OH in one-pot cascade catalysis makes it a superior bifunctional heterogeneous catalyst (Table S5†) and illustrates its conceptual feasibility.

## Conclusion

In summary, the present study reports the successful fabrication of an effective heterogeneous catalyst achieved *via* strategic modifications post-synthesis of the polymer. The developed catalyst, 120-MI@OH, features antagonistic catalytic sites that exist in close proximity whilst being spatially isolated. Owing to the presence of acidic as well as basic catalytically active sites, the polymer catalyst displayed outstanding performance in promoting the cyanosilylation (acid catalyzed) and Knoevenagel condensation (base catalyzed) under mild conditions. Additionally, the presence of antagonistic sites enabled 120-MI@OH to efficiently catalyze the one-pot deacetalization-Knoevenagel condensation cascade reaction. The catalyst retained its efficiency following 6 cycles of a reusability test, whilst it was also effective at large scale. Furthermore, the practical relevance of the catalyst was established by processing it into composite beads that were used to promote the cascade catalysis under continuous flow conditions without compromising activity. This work may trigger the strategic development of effective heterogeneous catalysts based on function-led porous polymer catalysts that have structures that are easily modified and are also cost-effective.

## Data availability

All the experimental data supporting this article has been uploaded as part of the ESI.†

## Author contributions

S. L. conceived the project idea. S. L. and G. K. D. designed the experiments. S. L. synthesized and characterized the materials. S. L. and G. K. D. analyzed the data. All the catalytic tests were performed by S. L. and G. K. D. S. F. acquired and analyzed the XPS data. Composite bead preparation was carried out by S. L. and S. F. G. K. D. was responsible for the physisorption measurements. S. F. did the graphical artwork. S. L. wrote the paper while all the authors contributed to the revising of the manuscript and gave their approval of the final version. The whole project was supervised by S. K. G.

## Conflicts of interest

There are no conflicts to declare.

## Supplementary Material

SC-014-D3SC03525E-s001
