# Supplementary material for: Organic porous heterogeneous composite with antagonistic catalytic sites as a cascade catalyst for continuous flow reaction
Source: Chem Sci. 2023 Sep 7;14(38):10591–601. doi: 10.1039/d3sc03525e (PMC10548525; doi:10.1039/d3sc03525e)
Supplement: SC-014-D3SC03525E-s001 [file SC-014-D3SC03525E-s001.pdf]

Electronic Supplementary Material (ESI) for Chemical Science.
This journal is © The Royal Society of Chemistry 2023

Supporting Information

All Organic Porous Heterogeneous Composite with Antagonistic Sites as Cascade Catalyst for Continuous Flow Reaction

Sumanta Let, [a] Gourab K. Dam, [a] Sahel Fajal, [a] and Sujit K. Ghosh *[a,b]

[a] Department of Chemistry, Indian Institute of Science Education and Research Pune, Dr. Homi Bhabha Road, Pashan, Pune 411008, India. E-mail: sghosh@iiserpune.ac.in.

[b] Centre for Water Research (CWR), Indian Institute of Science Education and Research (IISER) Pune, Dr. Homi Bhaba Road, Pashan, Pune 411008, India.

Corresponding author e-mail: sghosh@iiserpune.ac.in

Table of contents

Section S1

Materials, General Characterizations and Physical Measurements ………………….…..…..S3-S4

Section S2

Synthesis procedures……………………………………………………..…………...S5-S7

Section S3

General experimental procedures…..…………………………………………………..S8-S10

Section S4

Structural & morphological characterizations, Results…………………….…..……...……S11

Section S5

^{1}H NMR of Products……..…………………………………….…………………..….S37

Section S6

^{1}H NMR of continuous flow cascade catalysis..……………………………………..….S37

Section S1: Materials, General Characterizations and Physical Measurements

S1.1. Materials: Starting materials, Benzyl chloride, $FeCl_3$, formaldehyde dimethyl acetal (FDA), 1-Methylimidazole, were purchased from Sigma-Aldrich. NaOH, THF, 1,2-Dichloroethane (DCE) and remaining solvents were obtained locally. These chemicals were used without further purification.

S1.2. General Characterizations and Physical Measurements:

S1.2.1 Fourier transform infrared spectroscopy (FT-IR): The IR spectra were recorded using NICOLET 6700 FT-IR spectrophotometer using KBr pellet in 550-4000 cm^{-1} range.

S1.2.2 Thermogravimetric analysis (TGA): Thermogravimetric analyses were performed using Perkin-Elmer STA 6000 TGA analyzer by heating the samples from 40 to 800 ^{o}C under N_2 atmosphere with a heating rate of 10 °C min.

S1.2.3 Field emission scanning electron microscopy (FESEM): The morphology of the materials was investigated with a Zeiss Ultra Plus field-emission scanning electron microscope (FESEM) with an integral charge compensator and embedded EsB and AsB detectors (Oxford X-max instruments 80 mm^2 (Carl Zeiss NTS, GmbH). The elemental analysis was carried out using voltage of 15 KV equipped with an EDX detector. Data acquisition was performed with an accumulation time of >600s.

S1.2.4 Transmission electron microscopy (TEM): For high-resolution TEM analysis, all the samples were dispersed in isopropanol (0.5 mg/mL) and sonicated for 15 minutes. Then, the samples were left undisturbed for 2 minutes, and the upper part of the solution was taken for preparing TEM samples on a lacey carbon-coated copper grid (Electron Microscopy Science). TEM imaging and STEM-EDS were performed on the HRTEM (JEM-2200FS, JEOL) operating at acceleration voltage of 200 kV.

S1.2.5 Solid-state nuclear magnetic resonance (NMR) spectroscopy: Solid-state 13C cross-polarization-magic angle spinning (CP-MAS) spectra were acquired on a Bruker 500 MHz NMR spectrometer with a CP-MAS probe. Carbon chemical shifts are expressed in parts per million (δ scale). The 13C chemical shifts were externally referenced to tetramethylsilane (δ = 0.0 ppm).

S1.2.6 Proton (^{1}H) nuclear magnetic resonance (NMR) spectroscopy: All ^{1}H spectra were recorded either on Bruker 400 MHz and Jeol 400 MHz spectrometers. The chemical shifts (δ) in ppm were referenced to the residual signal of deuterium solvents (^{1}H NMR $CDCl_3$). The multiplicities of the peaks are s (singlet), d (doublet), t (triplet), q (quartet), dd (doublet of doublet), m (multiplet).

S1.2.7 Powder X-ray diffraction (PXRD): Powder X-ray diffraction (PXRD) patterns were performed on a Bruker D8 Advanced X-ray diffractometer at room temperature using Cu Kα radiation (λ = 1.5406 Å) at a scan speed of 0.5 ° min^{-1} and a step size of 0.01° in 2θ.

S1.2.8 Nitrogen adsorption-desorption isotherm measurements: N_2 gas adsorption measurements were performed using BelSorp-Max instrument (Bel Japan). Prior to adsorption measurements, the activated samples were heated at 120 °C under vacuum for 12 hours using BelPrepvacII.

S1.2.9 X-ray photoelectron spectroscopy (XPS): As-obtained powder samples was stuck to conductive paste and then measured by X-ray photoelectron spectroscopy using K-Alpha+ model (Thermo Fischer Scientific, UK) with Al Kα source.

S1.2.10 Temperature-programmed desorption (TPD) measurements: The temperature programmed desorption (TPD) study was done using CHEMBET™ TPR/TPD, QUANTACHROME.

Section S2: Detail synthetic procedures of HCP120-Cl, HCP 120-MI@Cl and HCP 120-MI@OH

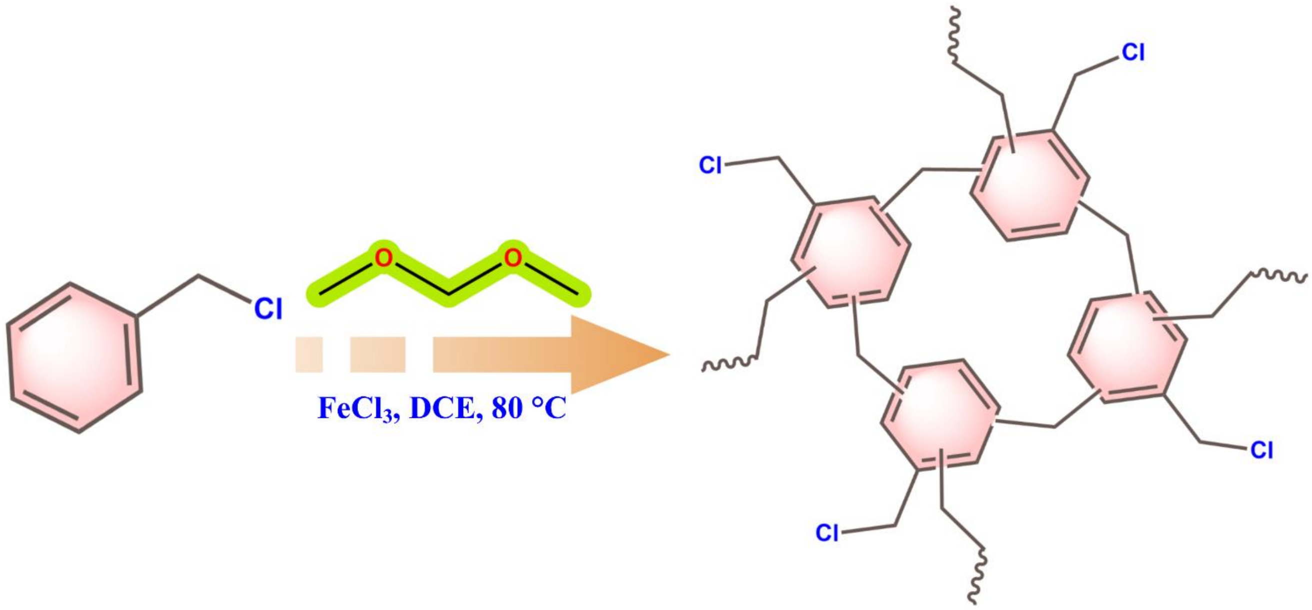

Scheme S1: Synthesis of HCP120-Cl

S2.1 Synthesis of HCP120-Cl: HCP120-Cl was synthesized by following our previous report (Scheme-1). [1]. Yield: 346 mg (~91%).

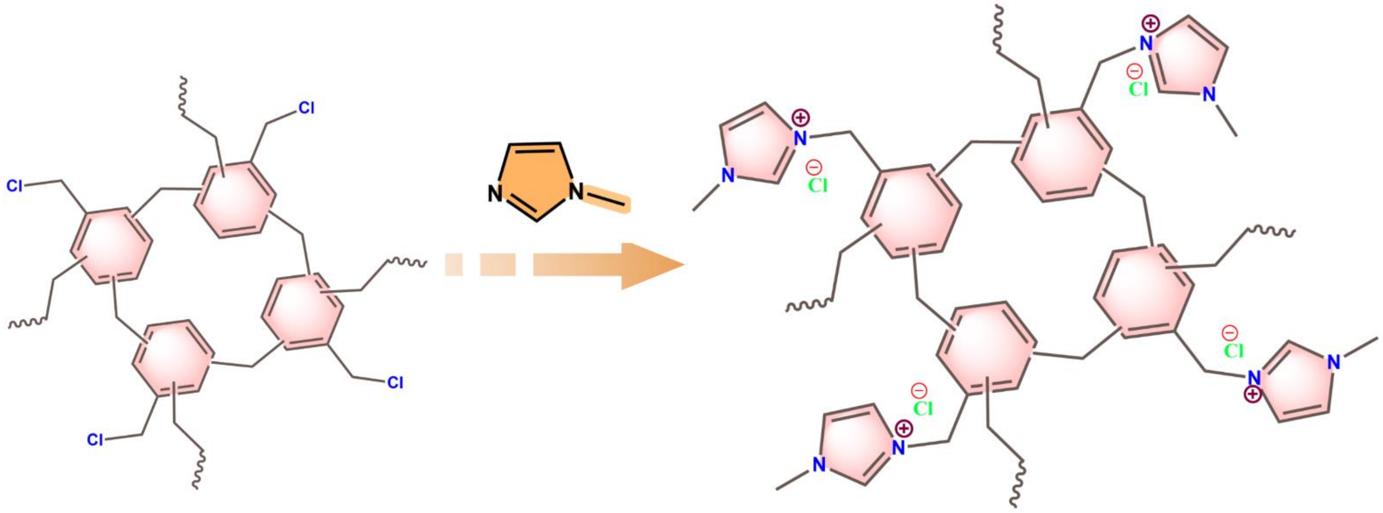

Scheme S2: Synthesis of HCP 120-MI@Cl

S2.2 Synthesis of HCP120-MI@Cl: Activated HCP 120-Cl (300 mg) was taken in a round bottom flask and to that 30 ml of acetonitrile (ACN) was added (Scheme-3). This was followed by dropwise slow addition of 3 mL 1-methyl imidazole (MI) and further allowed to react under stirring for 48 hrs. at reflux temperature. On completion of the reaction dark brown precipitate was filtered off and washed with copious amount of water for several times to remove excess MI. Then the precipitate was again washed with DMF, methanol, water, chloroform, dichloromethane and tetrahydrofuran (THF) repeatedly. Thus obtained dark brown colored solid material (HCP 120-MI@Cl) was subject to Soxhlet extraction in MeOH for 24 h to remove the high boiling solvents from the porous network of HCP 120-MI@Cl. The solvent exchanged phase was further heated at 140 °C under vacuum overnight to obtain the guest free activated material and with this phase further works have been carried out Yield: 352 mg (~ 92%).

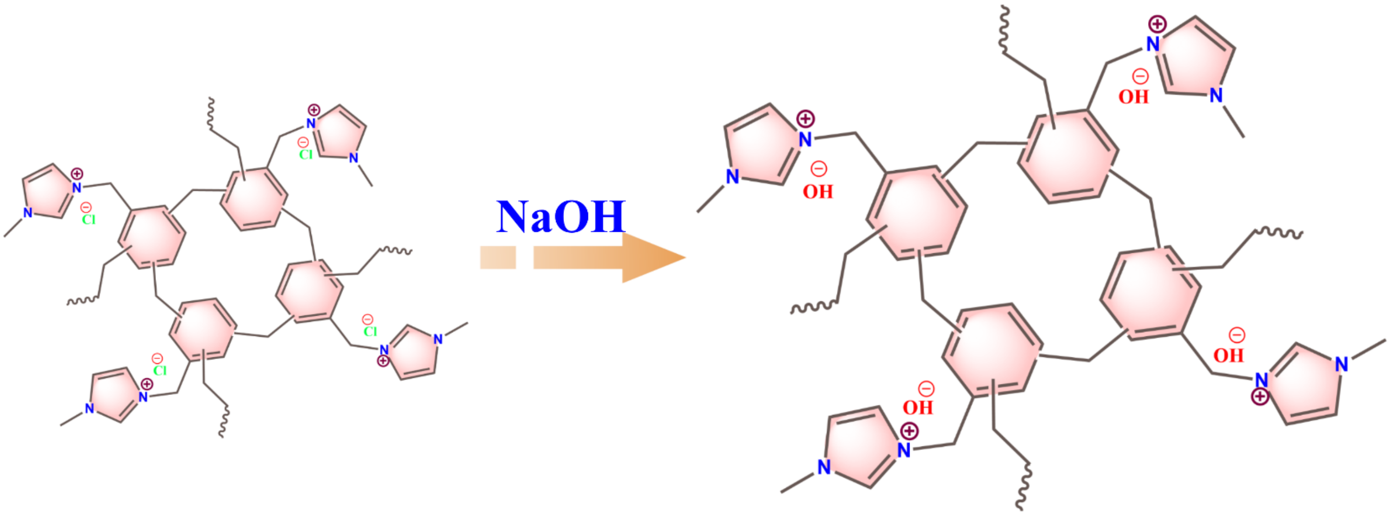

Scheme S3: Synthesis of HCP120-MI@OH

S2.3 Synthesis of HCP120-MI@OH: 100 mg of activated 120-MI@Cl and 250 mg NaOH were suspended in 10 mL H_2O. The resultant mixture was stirred at room temperature for 72 h. The mixture was then filtrated under reduced pressure and the obtained solid was washed with H_2O until neutral. Afterwards, the solid was dried in vacuo at 120 °C to yield 120-MI@OH (92 mg).

Section S3: General experimental procedures

S3.1 HCP 120-MI@OH catalyzed Knoevenagel Condensation:

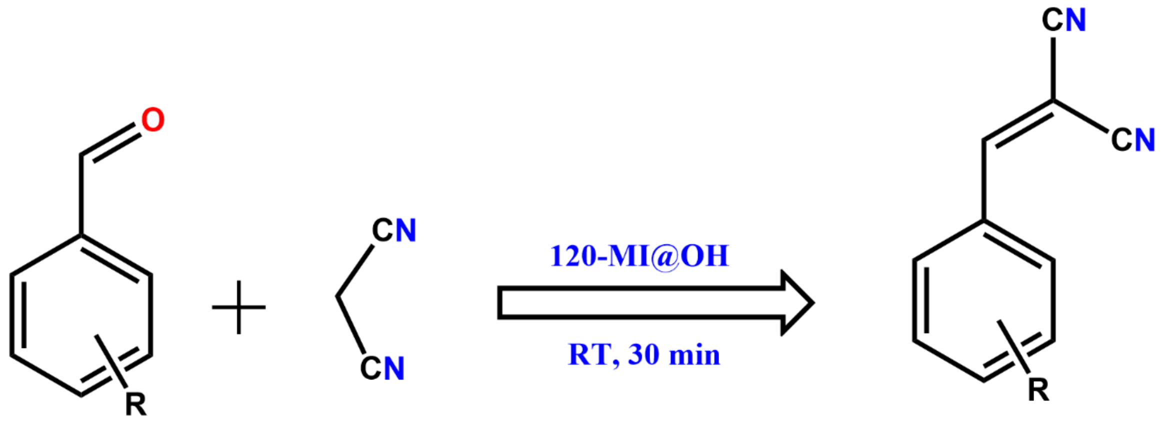

To a 10 mL reaction tube were sequentially added aldehyde (0.8 mmol, 1 equiv.), H_2O (4 mL), malononitrile (0.8 mmol, 1 equiv.), and the heterogeneous catalyst HCP 120-MI@OH (5 mg). The reaction mixture was then stirred at room temperature for 0.5 h. After extraction with CH_2Cl_2 (3 × 10 mL), the combined organic phase was dried over Na_2SO_4. After evaporation of the solvent under vaccum, the crude product was analyzed ^{1}H NMR using 1,3,5 trimethoxybenzene as the internal standard to determine product yield.

S3.2 HCP 120-MI@OH catalyzed Cyanosilylation:

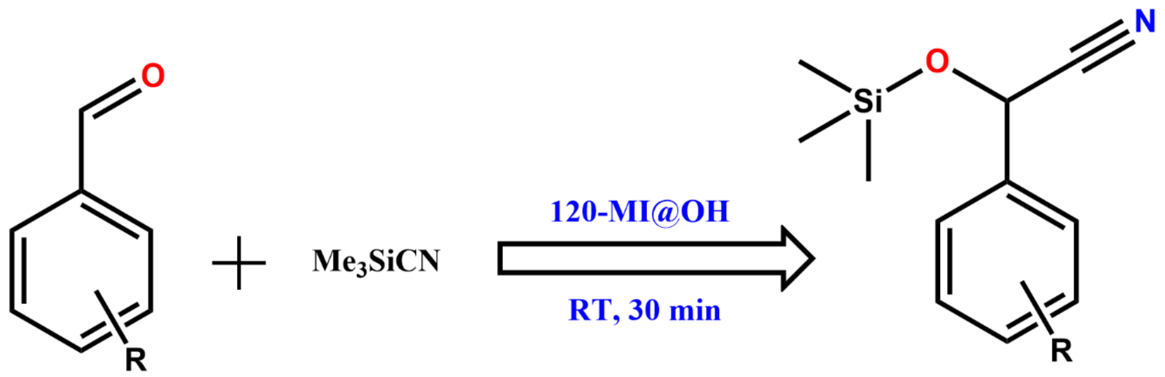

Me_3SiCN (2.0 mmol), carbonyl substrate (1.0 mmol) and catalyst 120-MI@OH (5 mg) were placed into a screw cap vial. The reaction mixture was vigorously stirred at room temperature for

0.5 h. After the reaction was completed, the crude product was extracted with CH_2Cl_2 (3 × 5 mL), and the solvent was evaporated. Finally, the conversion was determined by ^{1}H NMR using 1,3,5 trimethoxybenzene as the internal standard.

S3.3 HCP 120-MI@OH catalyzed One-Pot Cascade Catalysis Reaction:

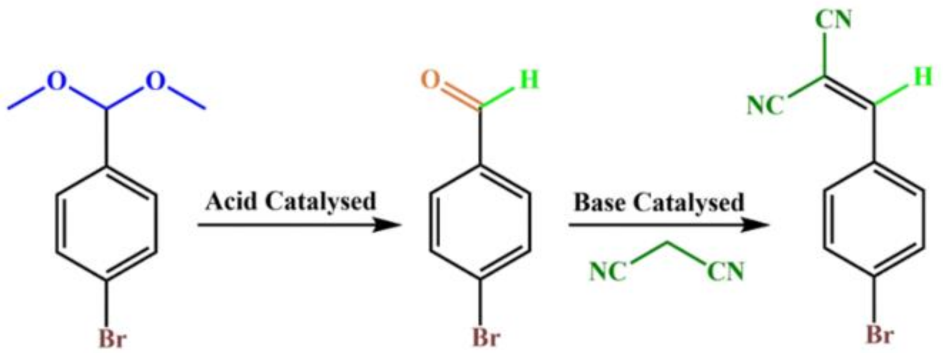

The one-pot deacetalization-Knoevenagel condensation reaction was performed in a 10 mL reaction tube equipped with a temperature controller and magnetic drive. A typical process is as follows: 5 mg of HCP 120-MI@OH catalyst together with a mixture of 4-bromobenzaldehyde dimethyl acetal (0.5 mmol), malononitrile (0.5 mmol) was put into the reactor. Then, 5 ml water (H_2O) was added as solvent and the reaction mixture was heated at 60 ^oC for 2 hrs. To test the reaction conversion, the compositions of starting reagent, intermediate product and the final product 3 were measured by ^{1}H NMR in $CDCl_3$. Comparing the integral of the phenylic proton from the final product benzylidene ethyl cyanoacetate (7.72 ppm) with the phenylic proton of the 4-bromobenzaldehyde dimethyl acetal (5.35 ppm) and 4-bromobenzaldehyde (9.96 ppm), the conversions of the starting reagent acetal and the intermediate product aldehyde were calculated by ^{1}H NMR using 1,3,5 trimethoxybenzene as the internal standard (see the example in Figure S15).

S3.4 Scaled-up Cascade Catalysis: The scaled up deacetalization-Knoevenagel condensation cascade catalysis was carried out by increasing the reactant, solvent as well as the catalyst amount. Typically, benzaldehyde dimethyl acetal (6.1 mL, 40 mmol), malononitrile (2.64

g, 40 mmol) and 150 mL water (H_2O) was added to a round bottom flask and was heated for 2 h at 60 °C. To test the reaction conversion, the formation of the final product and its purity ^{1}H NMR was performed in $CDCl_3$. Isolated product yield 5.54 g (89%)

S3.4 Continuous flow-through Cascade Catalysis:

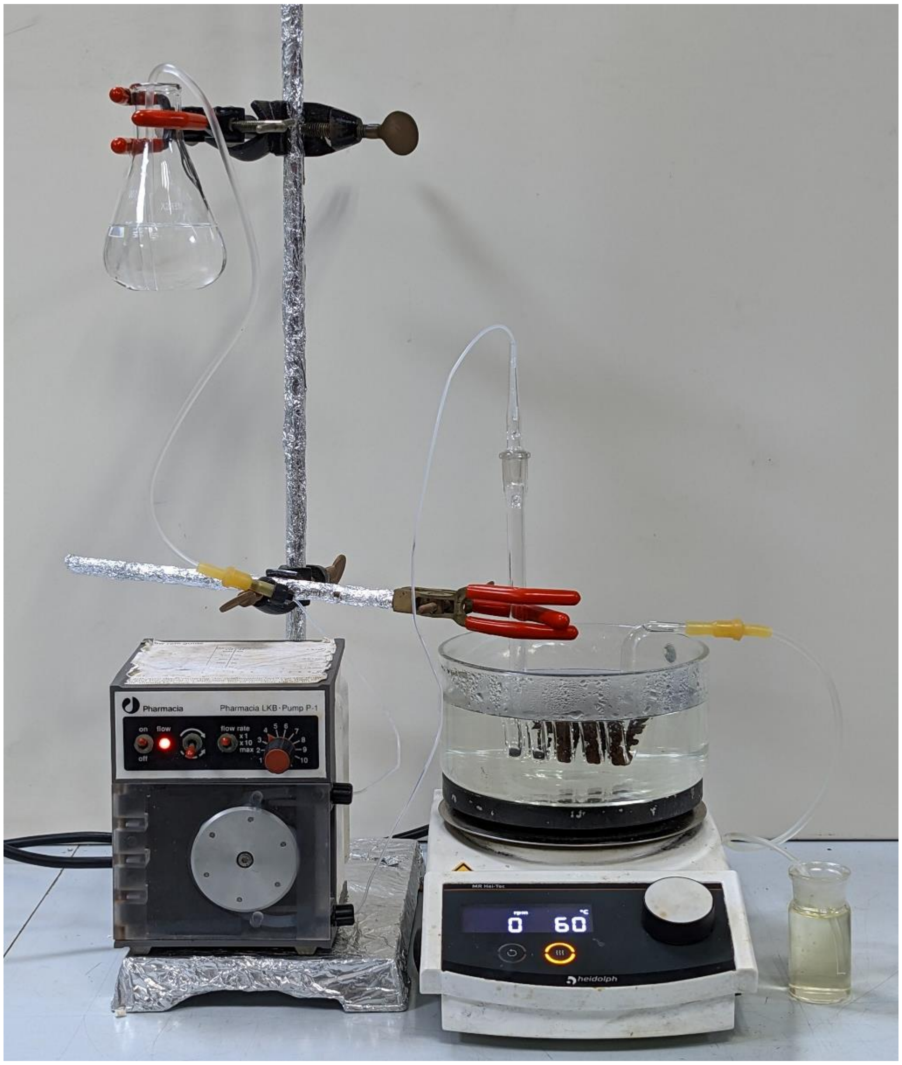

A mixture of benzaldehyde dimethylacetal (1.82 mL, 12.0 mmol), H_2O (5 mL), malononitrile (792.8 mg, 12.0 mmol) in C_2H_5OH solution (40 mL) was pumped through the spiral transparent glass tube reactor which is evenly charged with 120-MI@OH beads (0.2 g) at 60 °C with a flow rate of 0.4 mL min^{-1}.

Section S4: Structural and morphological characterizations

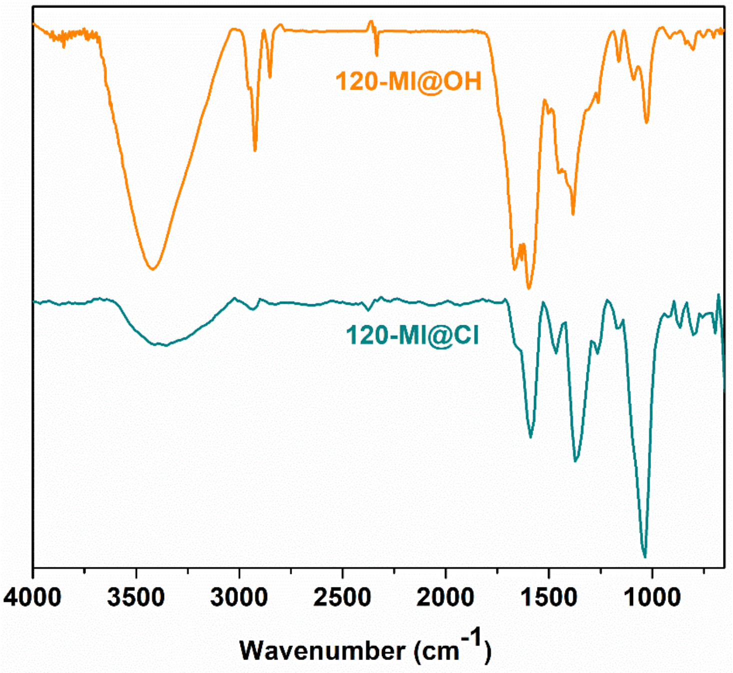

Figure S1: Infra-red (IR) spectra of 120-MI@Cl (cyan) and 120-MI@OH (orange).

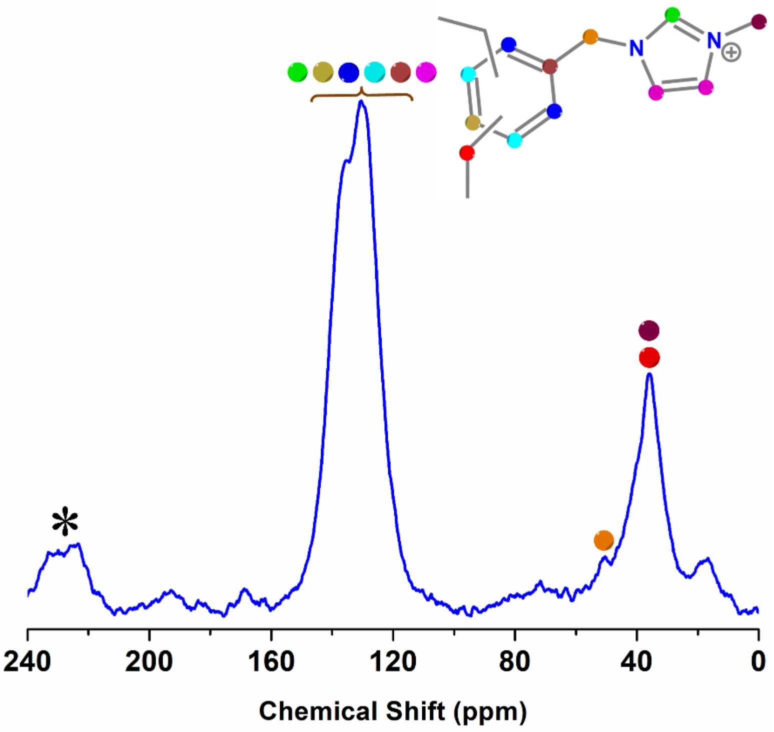

Figure S2: Solid-state ^{13}C CP-MAS NMR spectrum of polymer 120-MI@Cl.

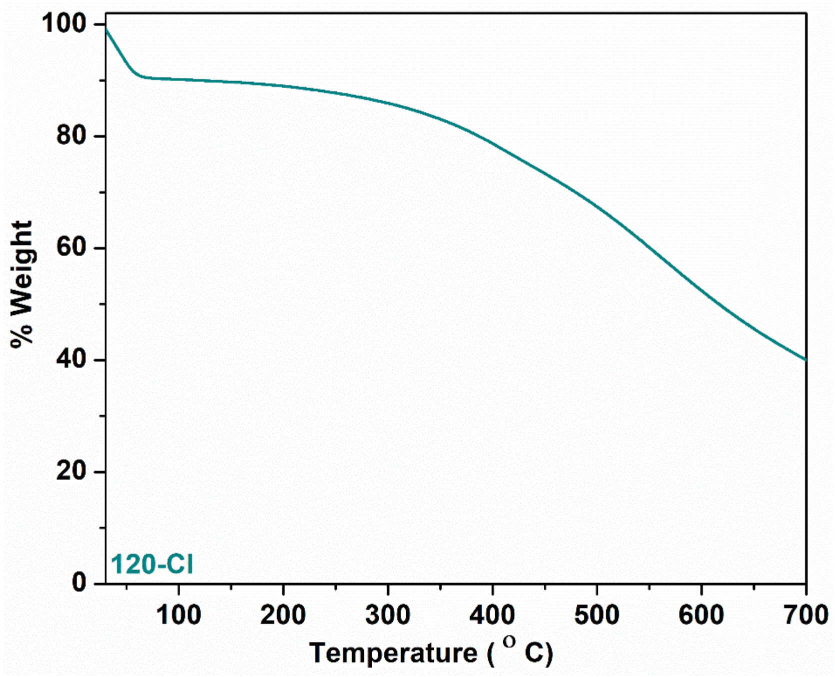

Figure S3: Thermogravimetric profile for pristine 120-Cl (cyan).

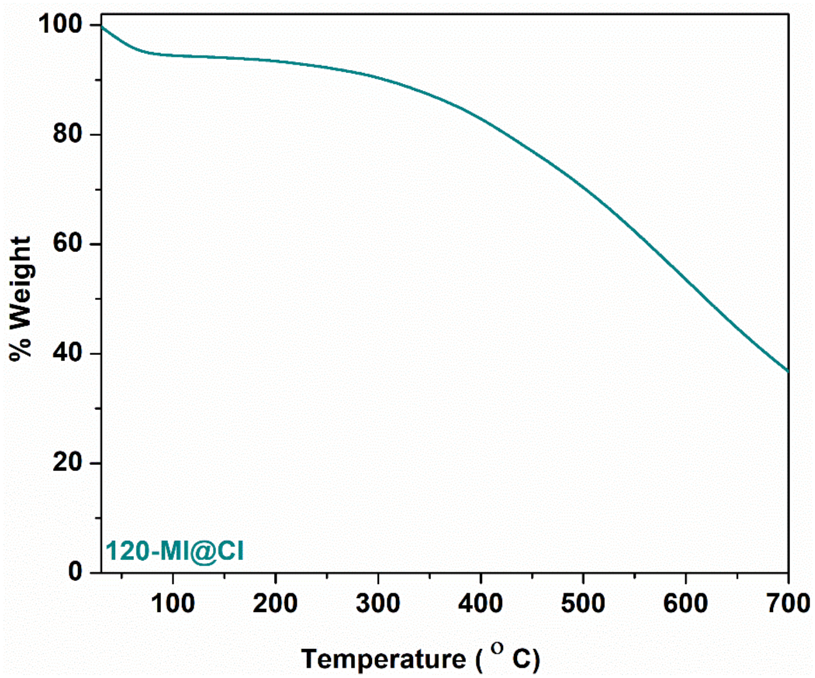

Figure S4: Thermogravimetric profile for pristine 120-MI@Cl (cyan).

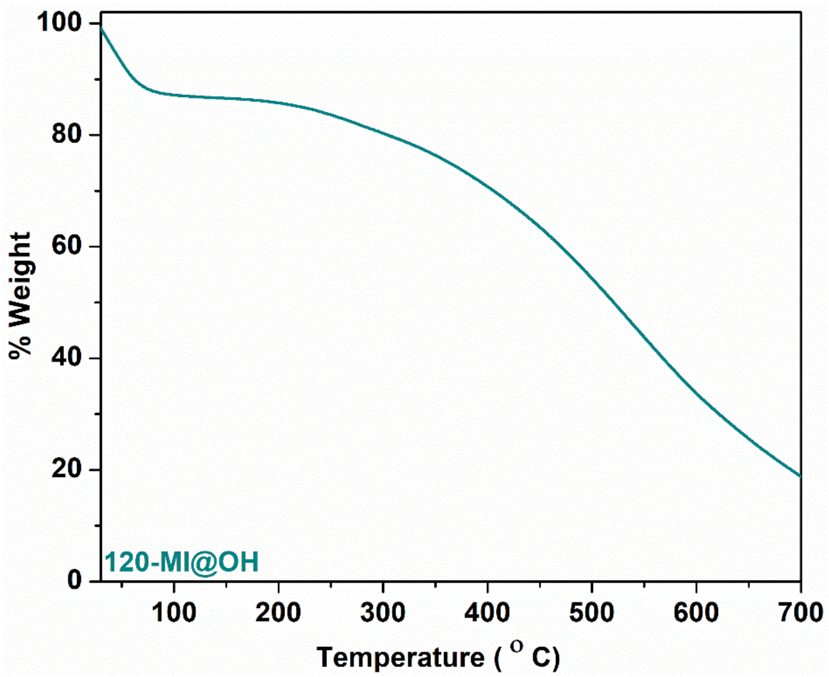

Figure S5: Thermogravimetric profile for pristine 120-MI@OH (cyan).

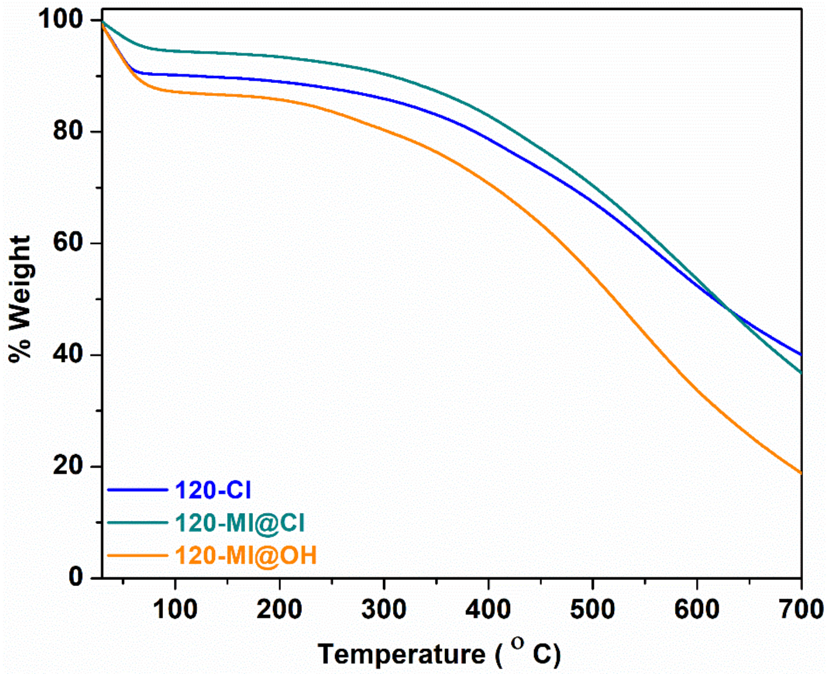

Figure S6: Thermogravimetric profile for pristine 120-Cl (blue), 120-MI@Cl (cyan) and 120-MI@OH (orange).

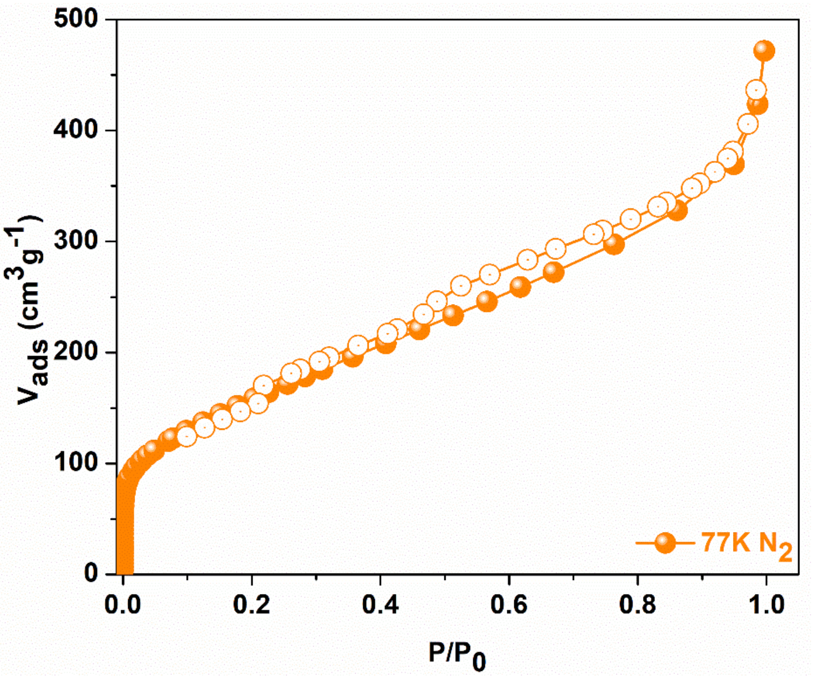

Figure S7: N_2 adsorption profile at 77 K for 120-MI@Cl

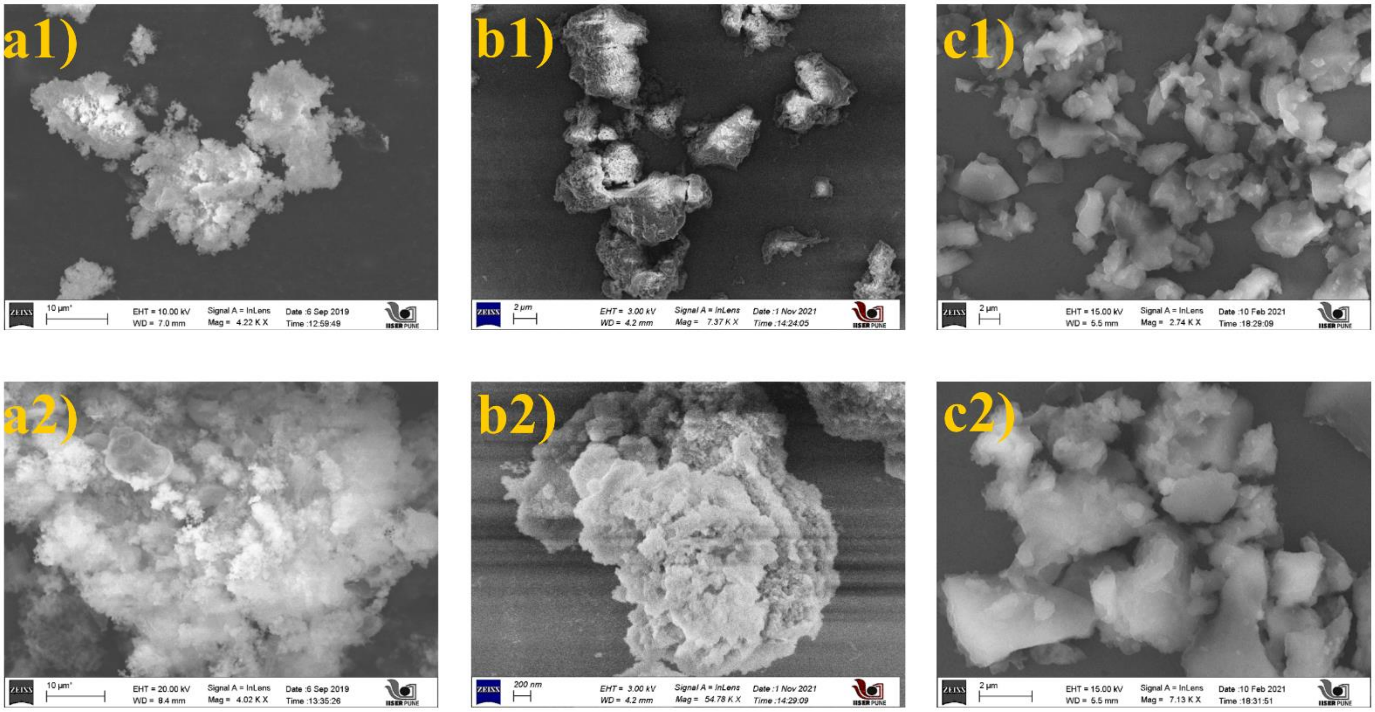

Figure S8: Magnified FESEM images of (a1-a2) 120-Cl, (b1-b2) 120-MI@Cl and (c1-c2) 120-MI@OH, scale bar = 1-2 µM

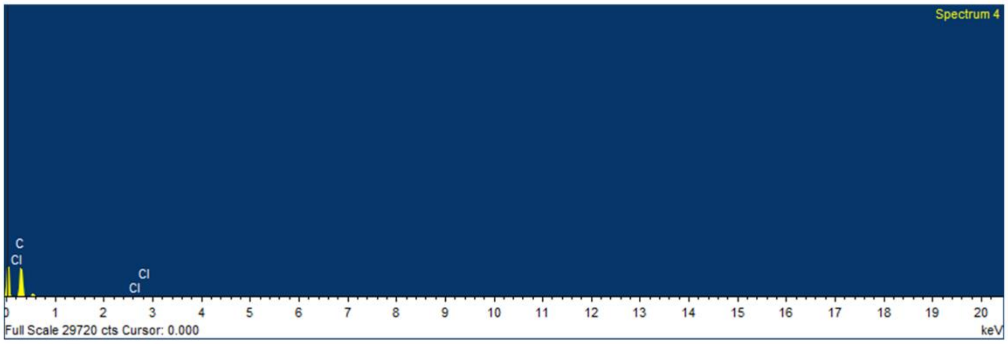

Element	Weight %
C	96.14
Cl	3.86

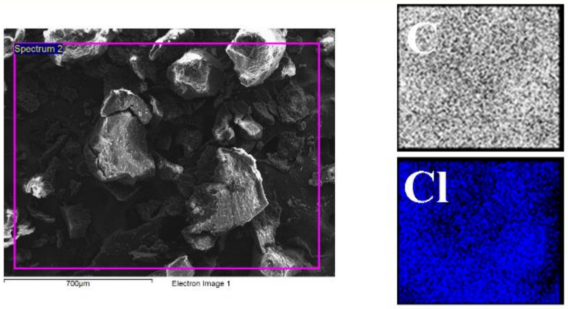

Figure S9: EDX analysis and elemental mapping of 120-Cl

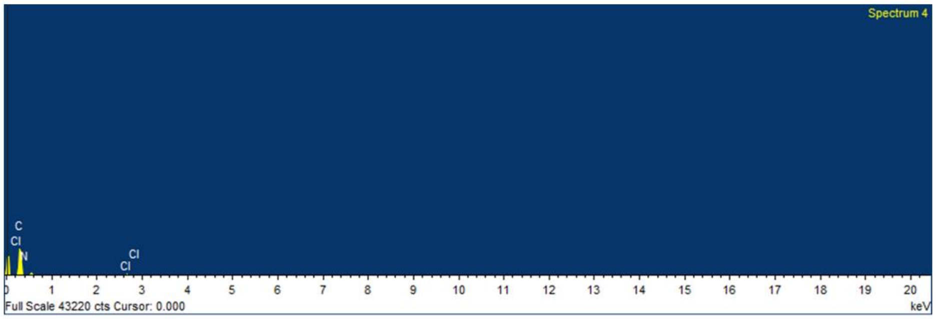

Element	Weight %
C	85.73
N	12.37
Cl	1.90

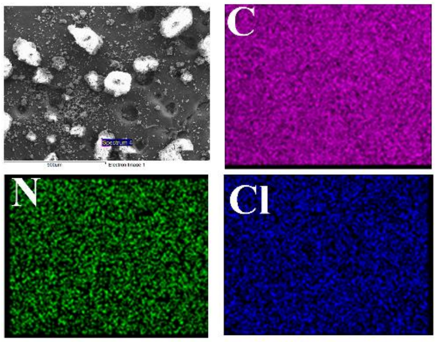

Figure S10: EDX analysis and elemental mapping of 120-MI@Cl

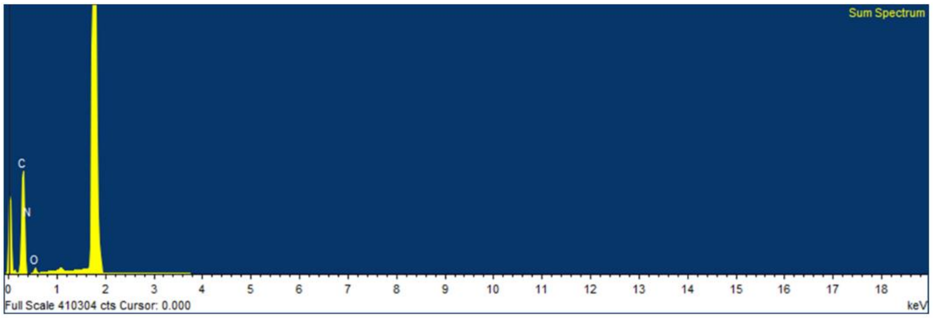

Element	Weight %
C	88.79
N	9.50
O	1.71

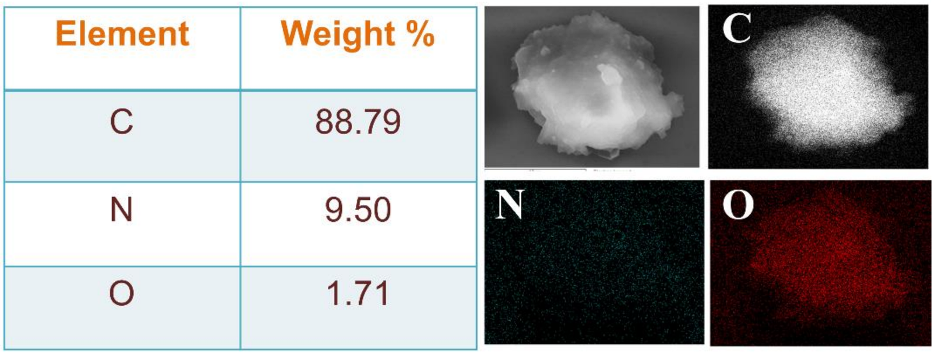

Figure S11: EDX analysis and elemental mapping of 120-MI@OH.

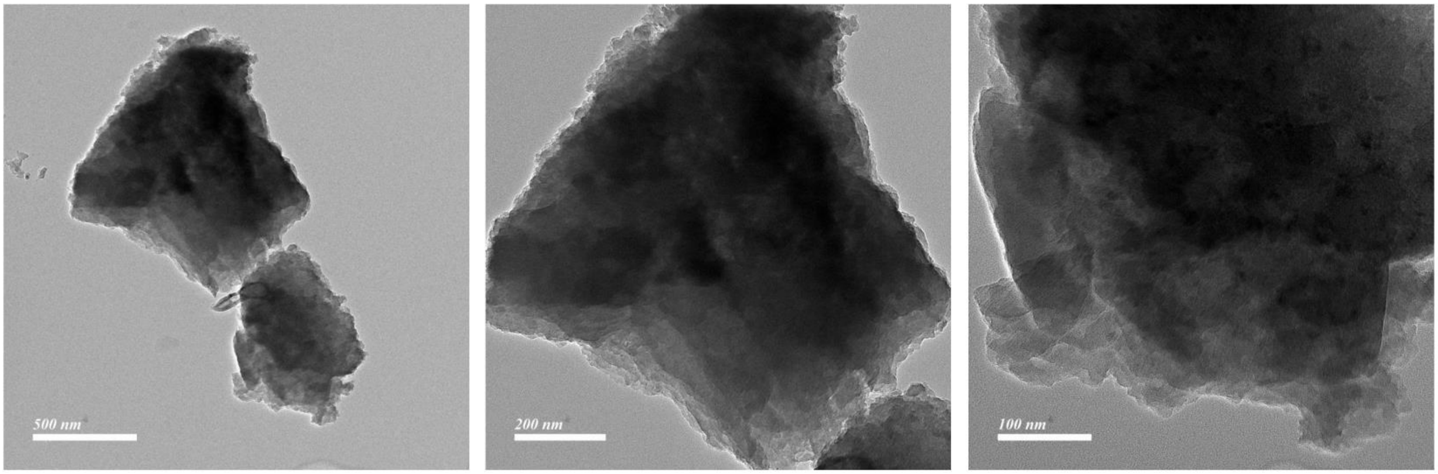

Figure S12: TEM images of 120-MI@OH.

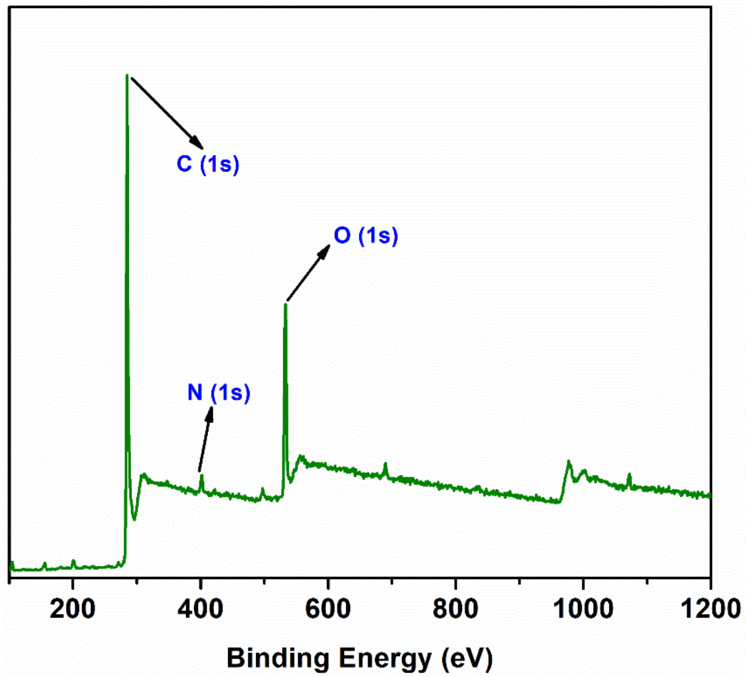

Figure S13: XPS survey scan spectra of 120-MI@OH.

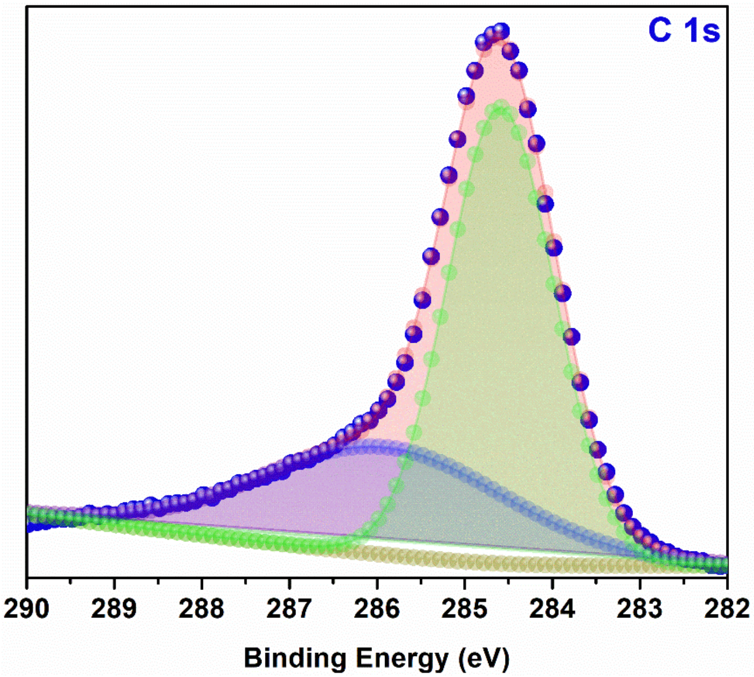

Figure S14: C 1s XPS spectrum of 120-MI@OH.

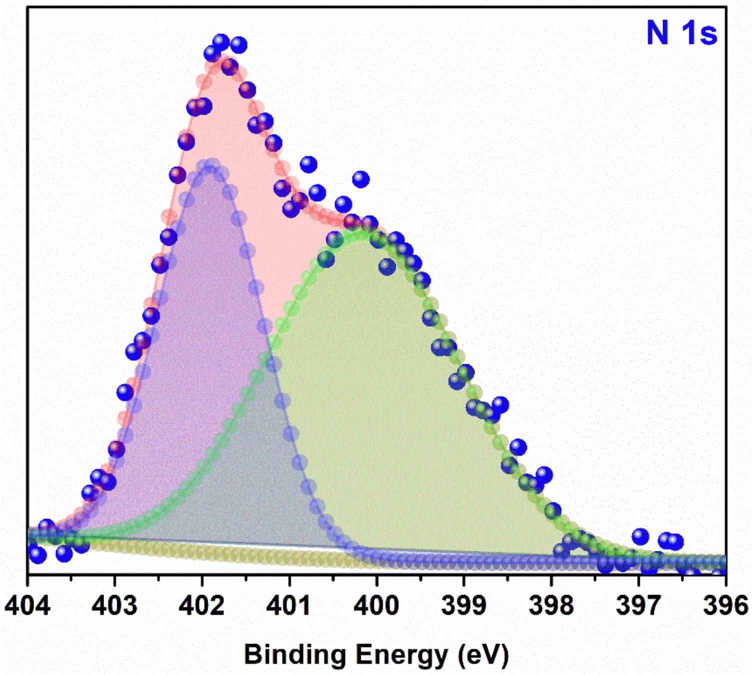

Figure S15: N 1s XPS spectrum of 120-MI@OH.

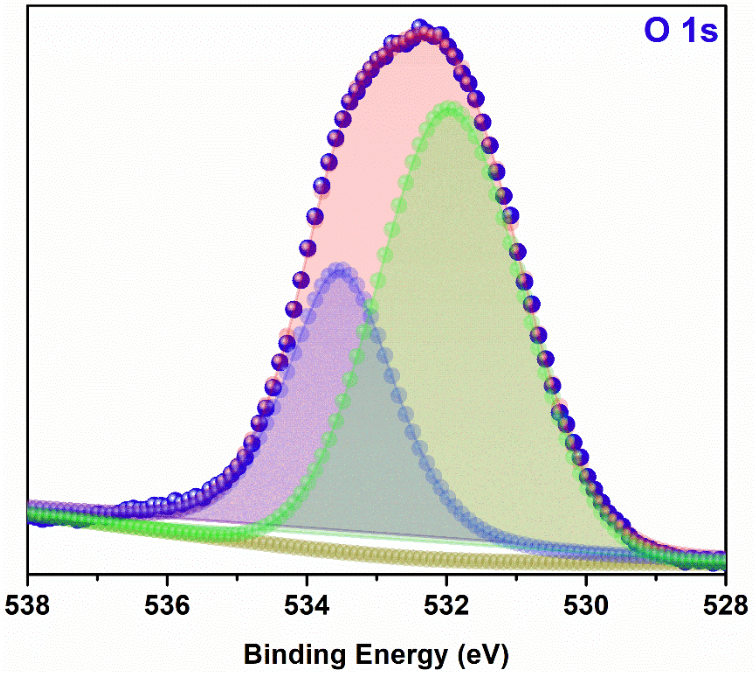

Figure S16: O 1s XPS spectrum of 120-MI@OH.

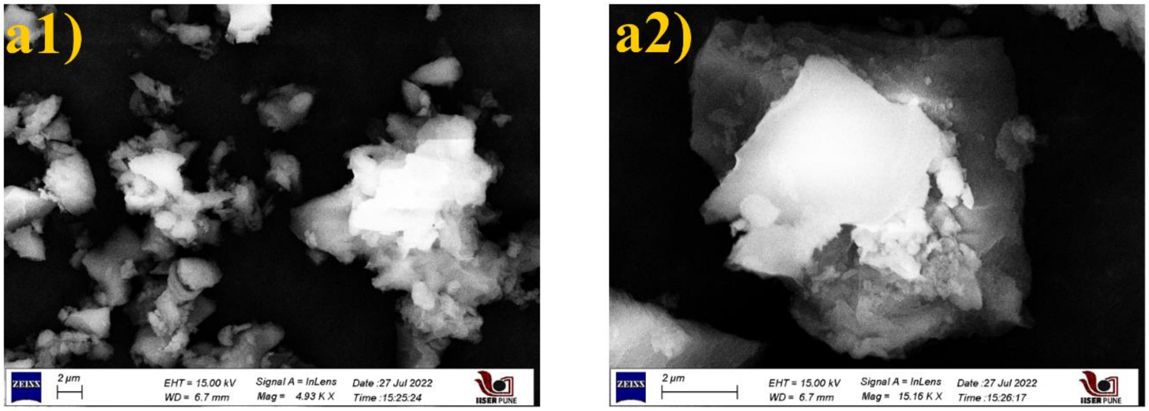

Figure S17: FESEM images of (a1-a2) spent120-MI@OH catalyst, scale bar = 2 µM

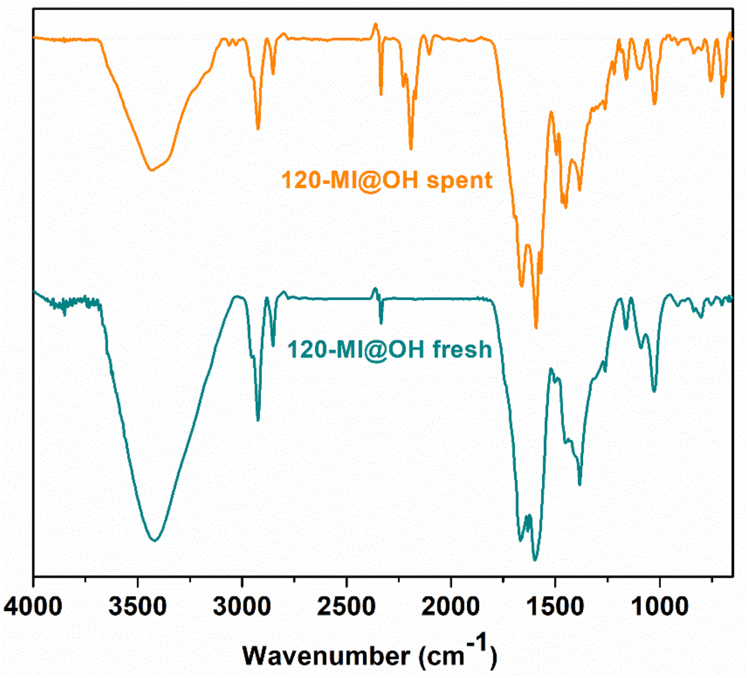

Figure S18: Infra-red (IR) spectroscopy of fresh120-MI@OH (cyan) and spent120-MI@OH (orange).

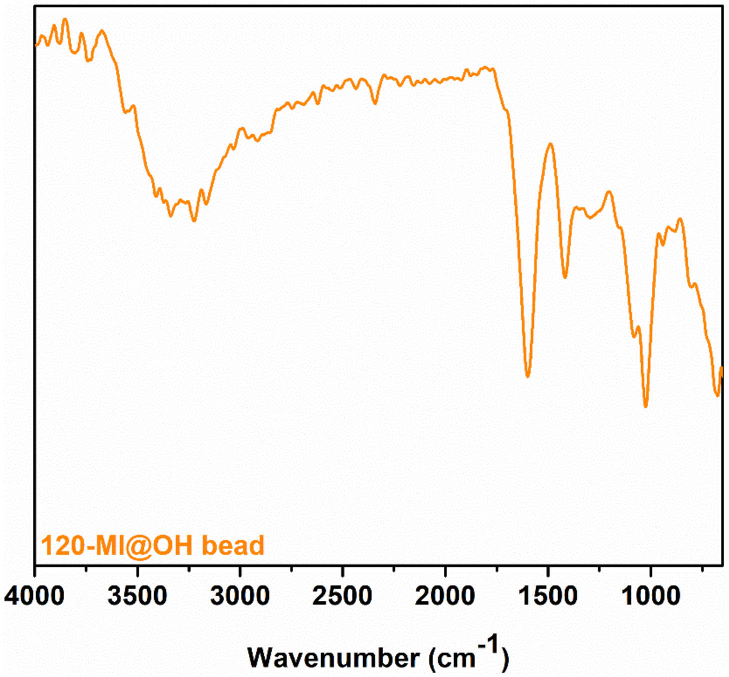

Figure S19: Infra-red (IR) spectroscopy of 120-MI@OH composite bead (orange).

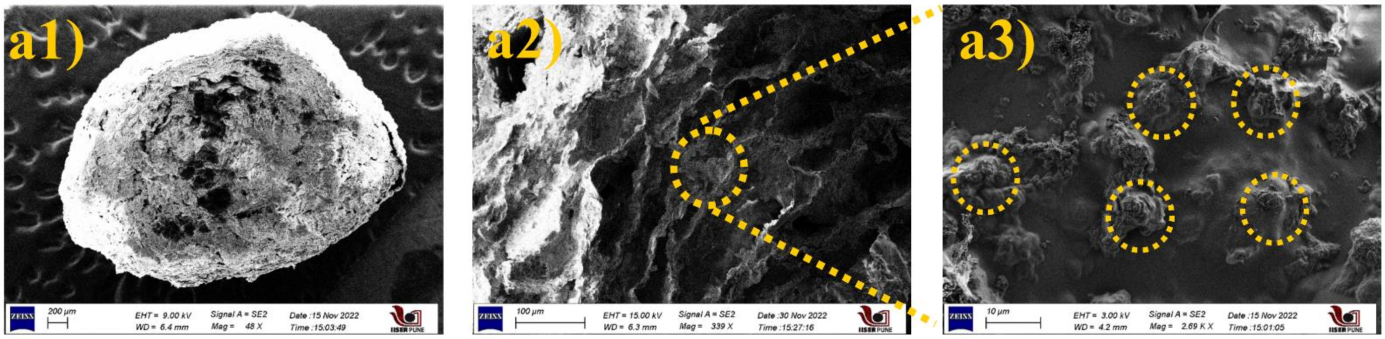

Figure S20: FESEM images of 120-MI@OH composite bead at different magnification showing the successful integration 120-MI@OH polymer inside the bead, scale bar = 200 µM (a1), 100 µM (a2) and 10 µM (a3)

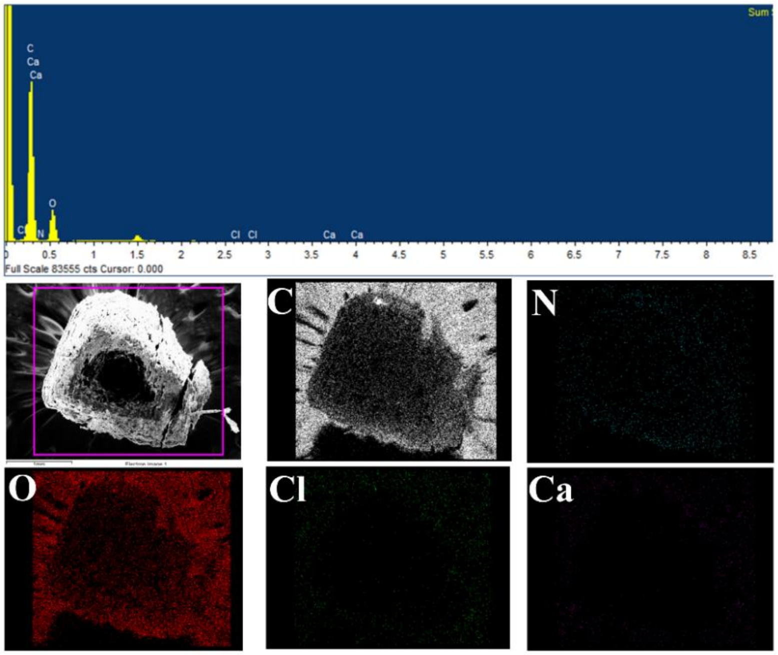

Figure S21: EDX analysis and elemental mapping of millimeter-sized 120-MI@OH composite bead.

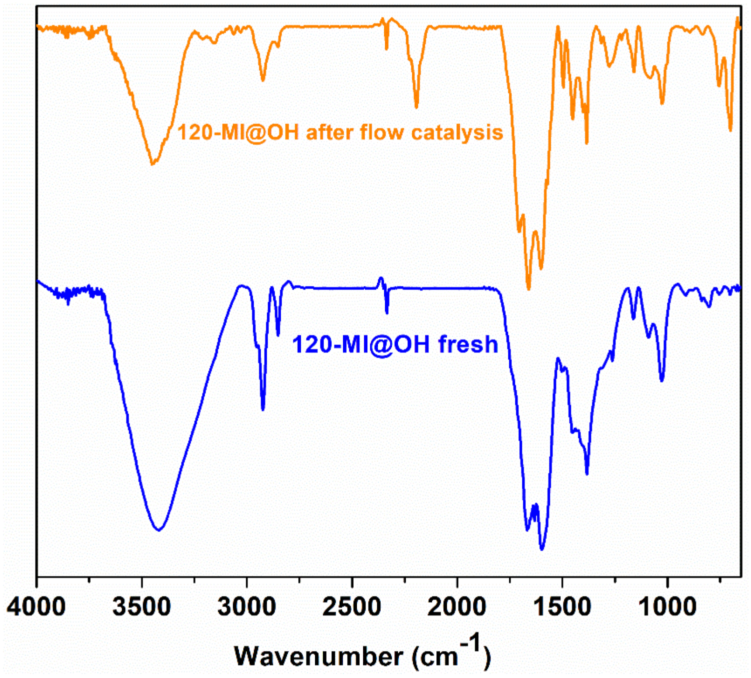

Figure S22: Infra-red (IR) spectroscopy of fresh120-MI@OH (blue) and 120-MI@OH after flow catalysis (orange).

.

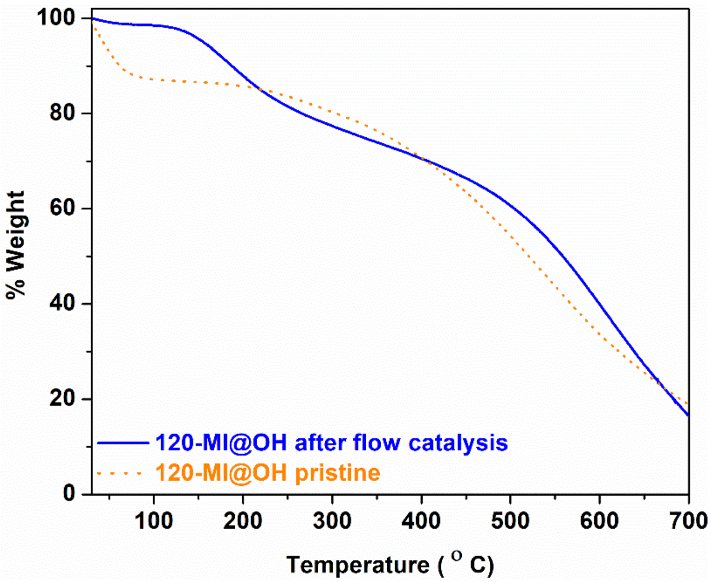

Figure S23: Thermogravimetric profile for pristine 120-MI@OH (orange) and 120-MI@OH after flow catalysis (blue).

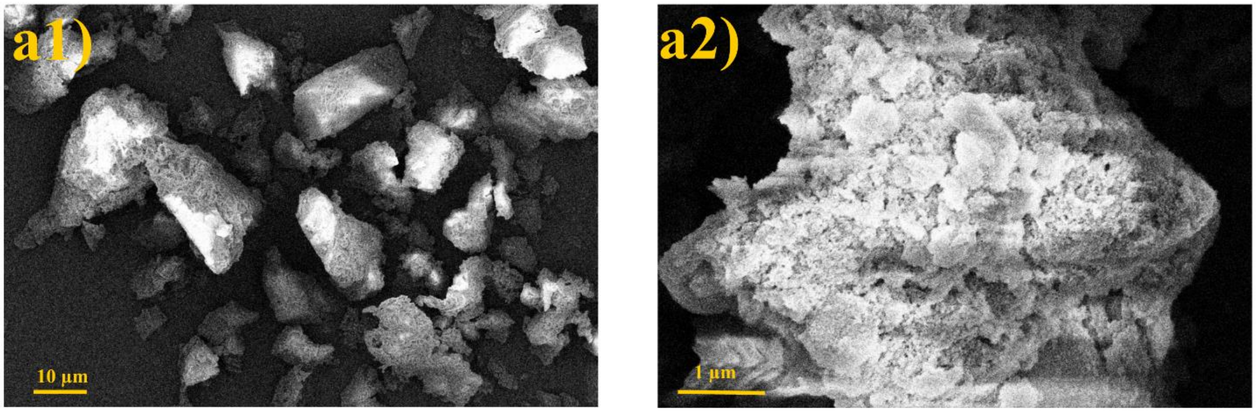

Figure S24: FESEM images of 120-MI@OH after flow catalysis.

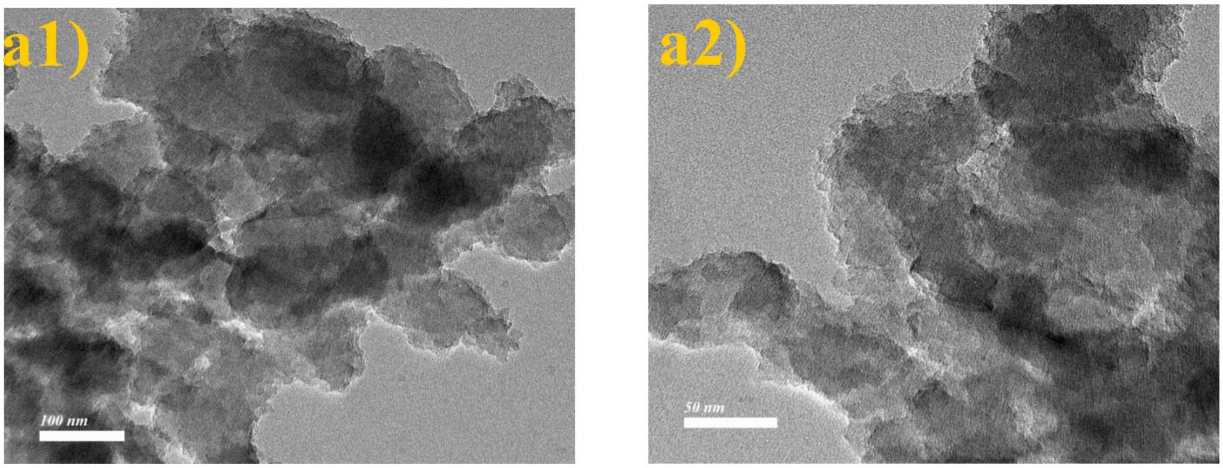

Figure S25: TEM images of 120-MI@OH after flow catalysis.

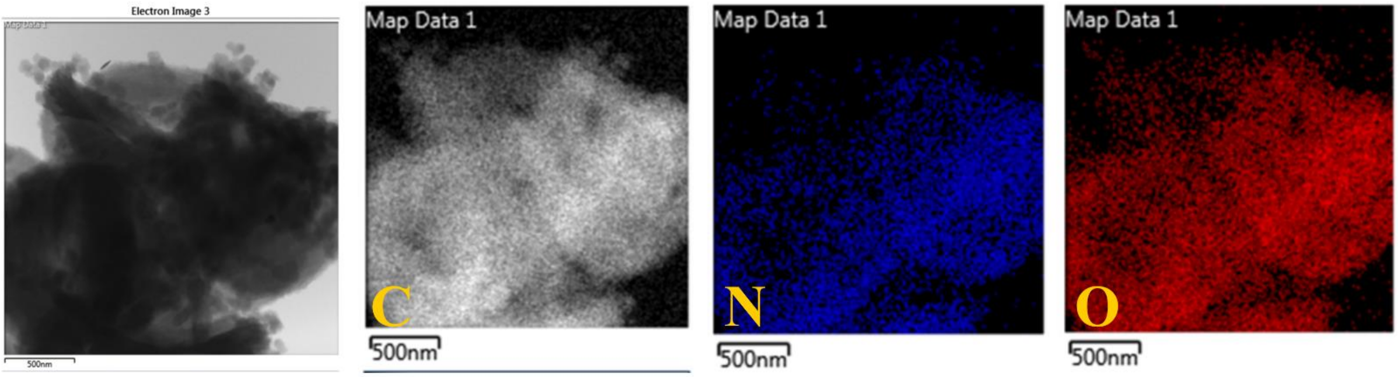

Figure S26: STEM–EDS mapping profile of 120-MI@OH after flow catalysis.

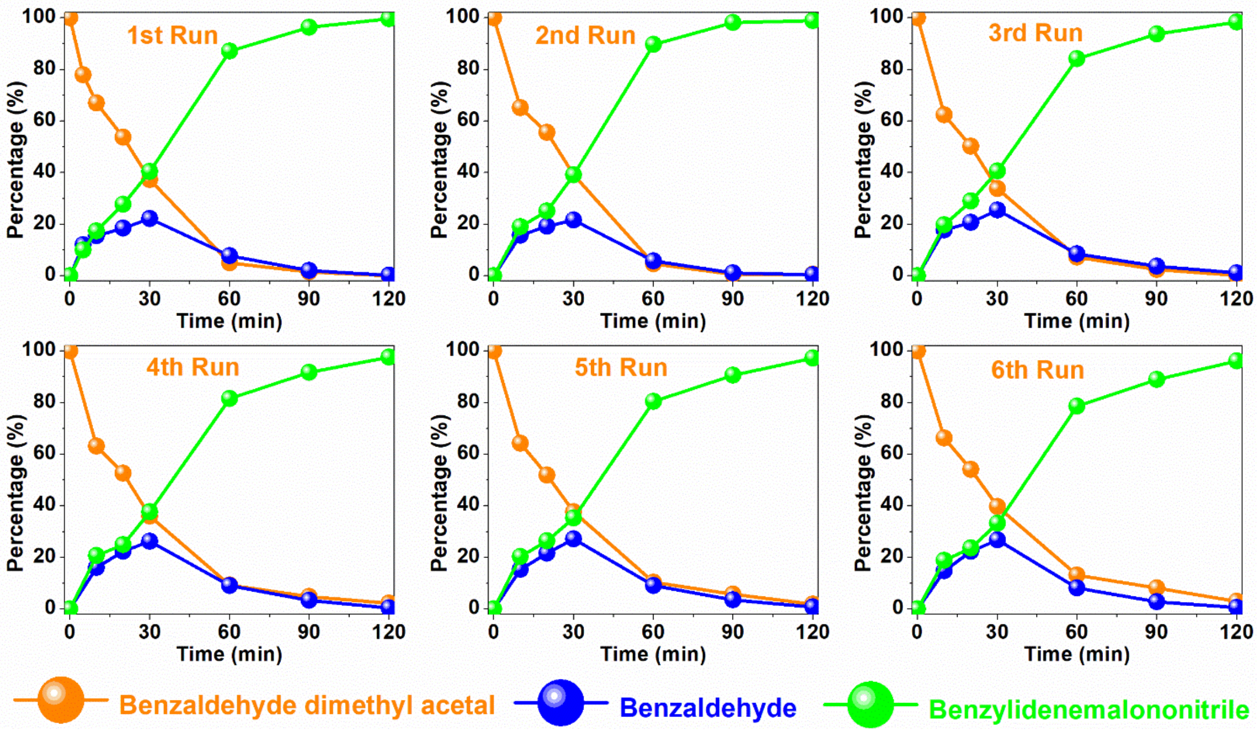

Figure S27: Kinetics monitoring of recyclable cascade catalytic reaction

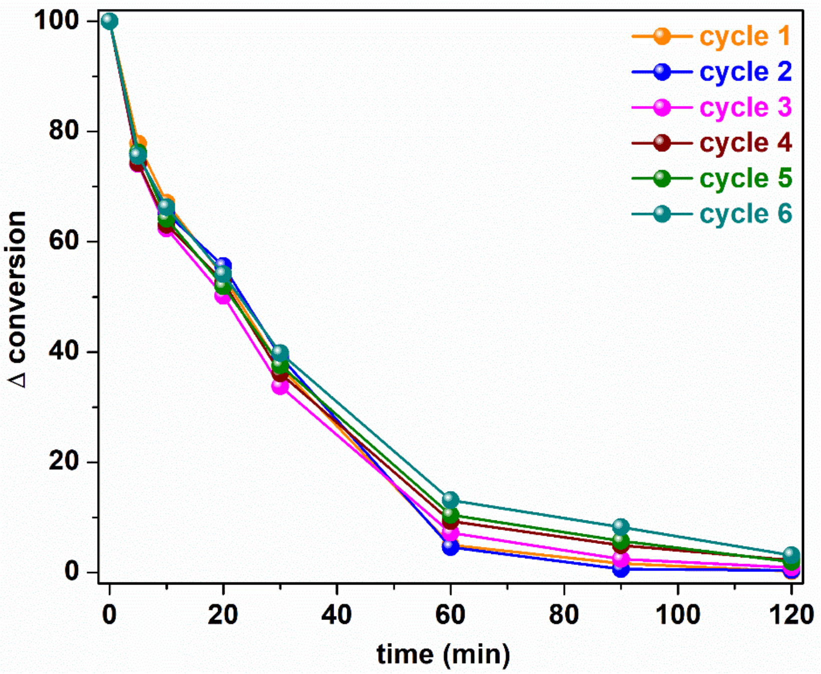

Figure S28: Δconversion vs time plot for different cycles of one-pot cascade catalysis by 120-MI@OH.

Table S1. Control experiments for 120-MI@OH catalyzed Knoevenagel condensation reaction

Entry	Catalyst	Solvent	Temp. (° C)	Time	Yield (%)[b]
1.	120-MI@OH (5 mg)	CH_2Cl_2	RT	1 h	56
2.	120-MI@OH (5 mg)	MeOH	RT	1 h	65
3.	120-MI@OH (5 mg)	H_2O	RT	1 h	>99
4.	120-MI@OH (5 mg)	H_2O	RT	0.5 h	>99
5.	120-MI@Cl (5 mg)	H_2O	80	2h	51
6.	No Catalyst	H_2O	RT	1h	6.8
7.	Supernatant Liquid of the H_2O suspension of 120-MI@OH	H_20	RT	1h	7.3

[a] Reaction conditions (Unless otherwise mentioned): a solution of the carbonyl compound (0.8 mmol, 1 equiv.), H_2O (4 mL), malononitrile (0.8 mmol, 1 equiv.), and the heterogeneous catalyst HCP 120-MI@OH (5 mg) was stirred at the indicated temperature for noted time duration. [b] Yield determined from ^{1}H-NMR.

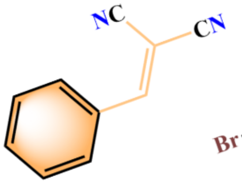

(1a, >99%)

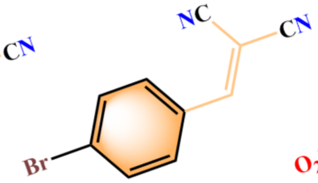

(1b, >99%)

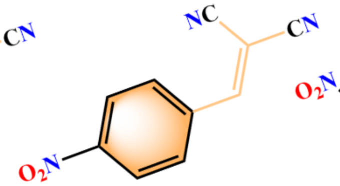

(1c, >99%)

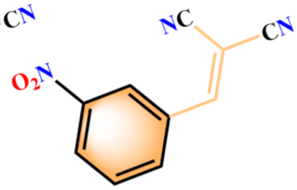

1d, (98%)

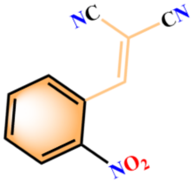

1e, (97%)

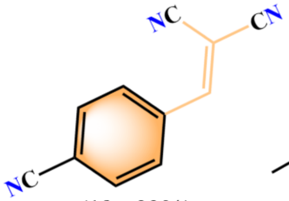

(1f, >99%)

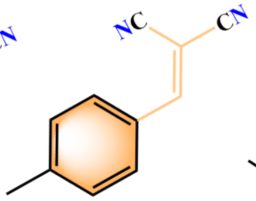

1g, (96%)

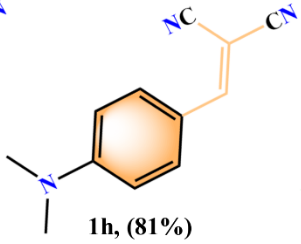

1h, (81%)

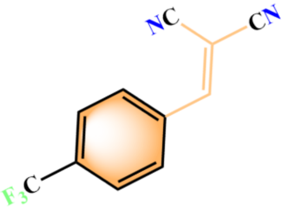

1i, (>99%)

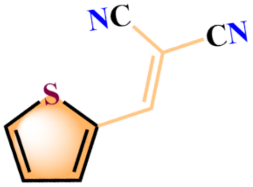

1j, (94%)

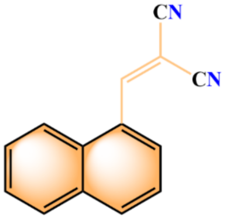

1k, (87%)

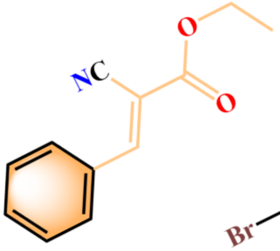

1l, (>99%)

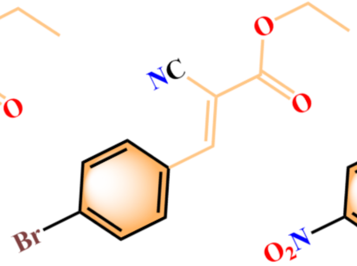

1m, (>99%)

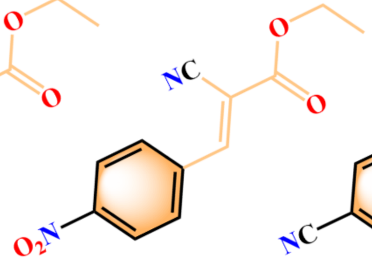

1n, (>99%)

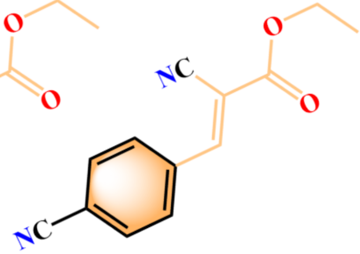

1o, (>99%)

Table S2. 120-MI@OH catalyzed Knoevenagel condensation reaction at room temperature. Conditions: 0.8 mmol of carbonyl compound, 0.8 mmol malononitrile/ ethyl cyanoacetate and 5 mg of catalyst was stirred in 4 mL H_2O for 0.5 h; Yield determined from ^1H NMR

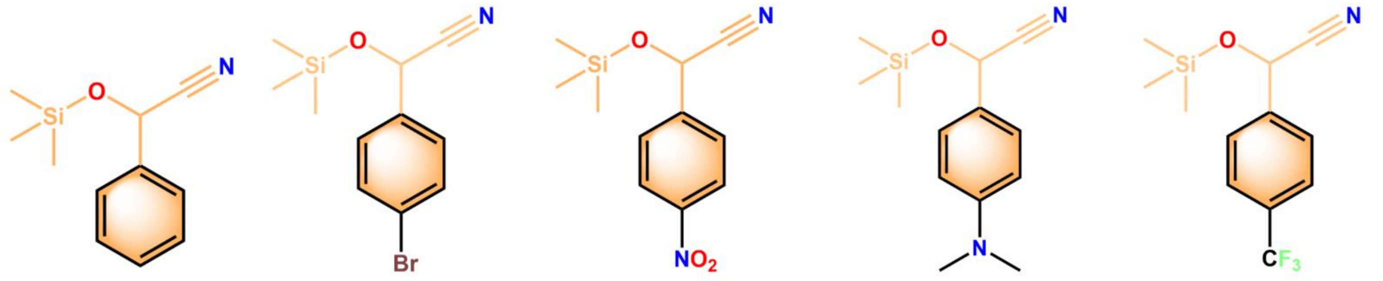

2a, (>99%) 2b, (>99%) 2c, (>99%) 2d, (93%) 2e, (>99%)

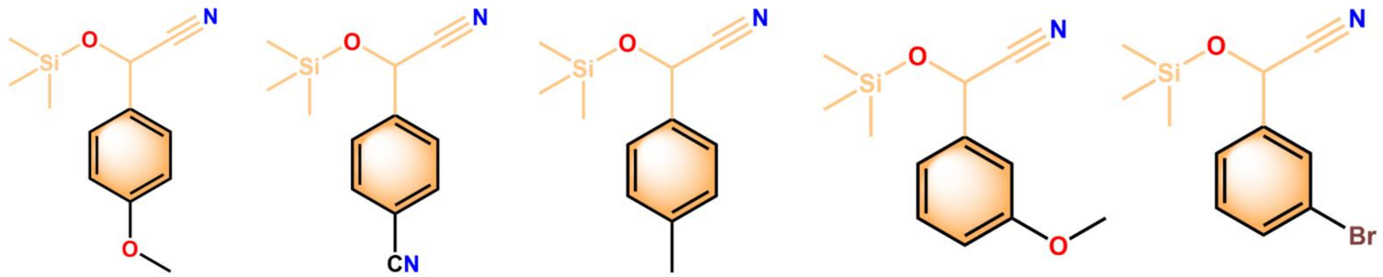

2f, (>99%) 2g, (>99%) 2h, (97%) 2i, (>99%) 2j, (>99%)

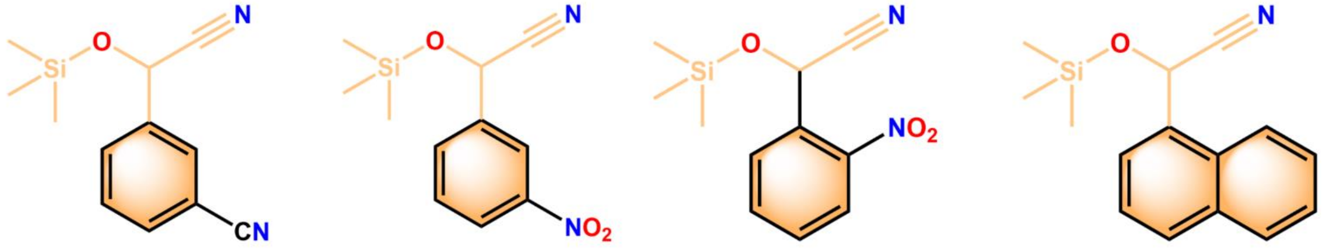

2k, (>99%) 2l, (>99%) 2m, (>99%) 2n, (>99%)

Table S3. 120-MI@OH catalyzed cyanosilylation reaction at room temperature. Conditions: Me_3SiCN (2.0 mmol), carbonyl substrate (1.0 mmol) and catalyst 120-MI@OH (5 mg) at RT for 0.5 h; Yield determined from ^1H NMR

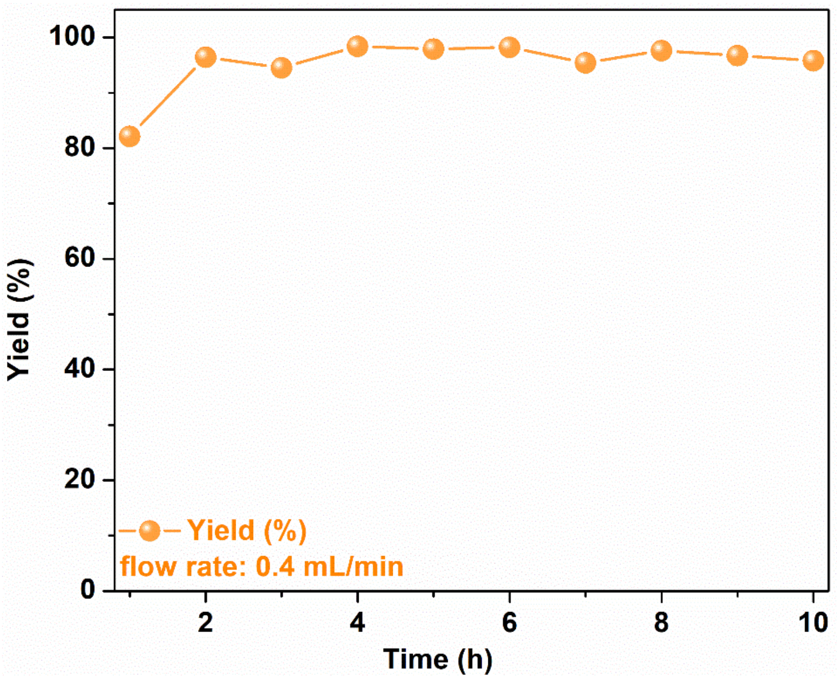

Figure S29: Yield over time of flow deacetalization-Knoevenagel condensation reactions under continuous flow condition.

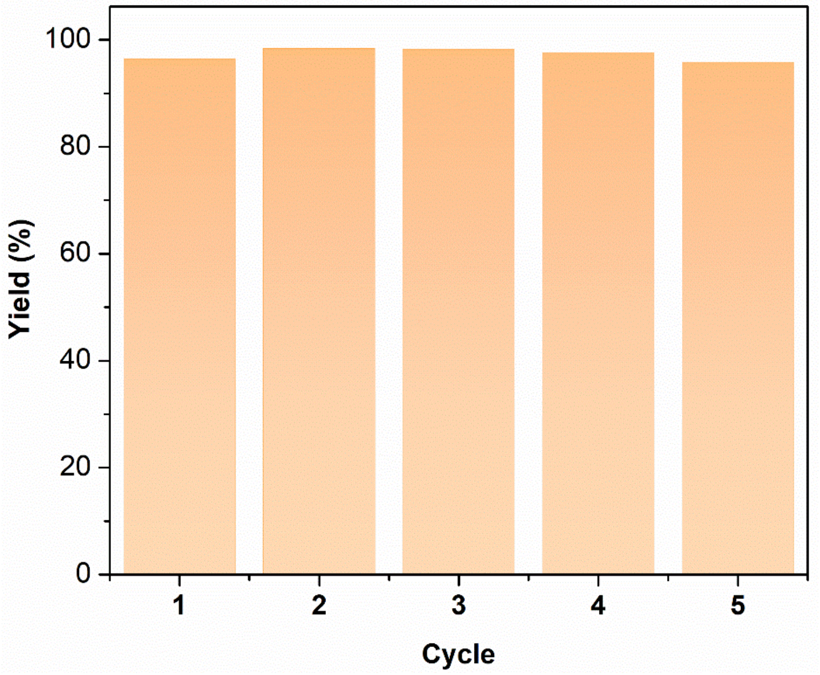

Figure S30: Recyclability test for one-pot cascade catalysis under flow.

Section S5: ^{1}H NMR spectra of compounds

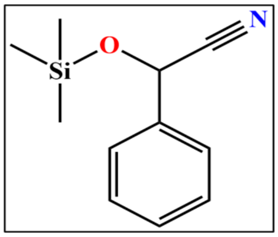

2-phenyl-2-((trimethylsilyl)oxy)acetonitrile:
^{1}H-NMR (400 MHz, $CDCl_3$, 25°C, TMS), δ (in ppm): 7.48 (d, 2H, ArH), 7.41 (d, J= 8 Hz, 3H, ArH), 5.50 (s, 1H, ArH), 0.24 (s, 9H, Alkyl H)

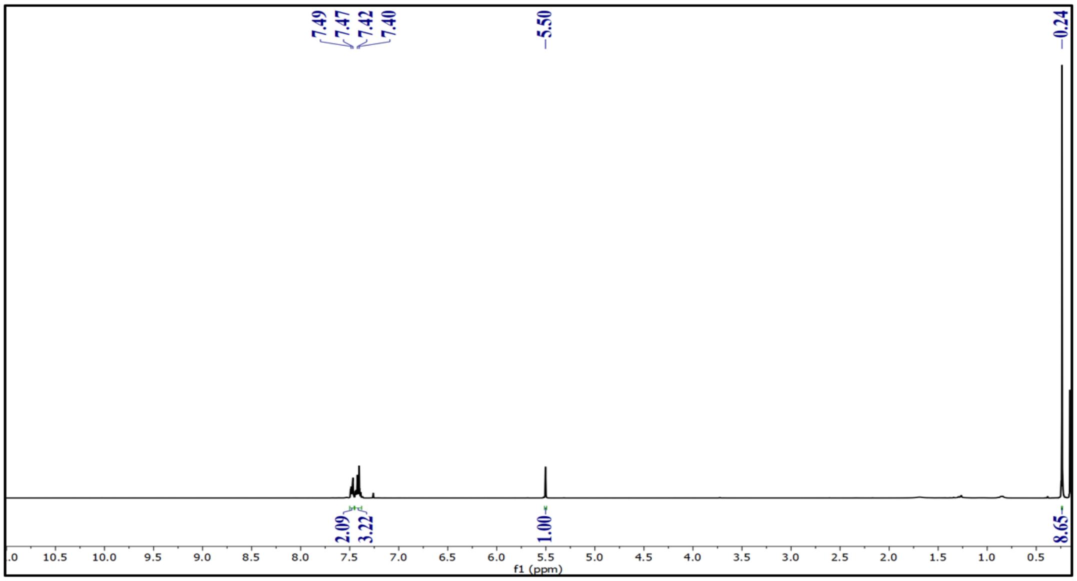

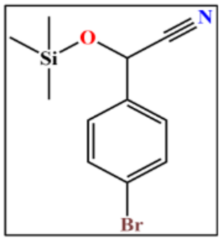

2-(4-bromophenyl)-2-((trimethylsilyl)oxy)acetonitrile:
^{1}H-NMR (400 MHz, $CDCl_3$, 25°C, TMS), δ (in ppm): 7.55 (d, J= 8 Hz, 2H, ArH), 7.35 (d, J= 8 Hz, 2H, ArH), 5.45 (s, 1H, ArH), 0.24 (s, 9H, Alkyl H)

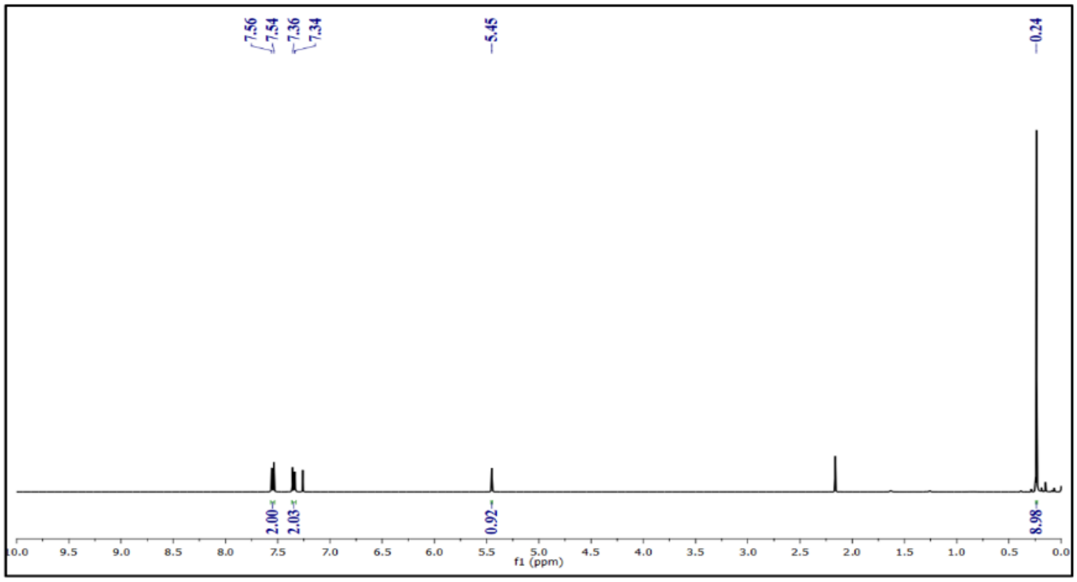

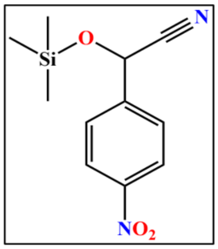

2-(4-nitrophenyl)-2-((trimethylsilyl)oxy)acetonitrile:
^{1}H-NMR (400 MHz, $CDCl_3$, 25°C, TMS), δ (in ppm): 8.28 (d, J= 8 Hz, 2H, ArH), 7.67 (d, J= 8 Hz, 2H, ArH), 5.60 (s, 1H, ArH), 0.28 (s, 9H, Alkyl H)

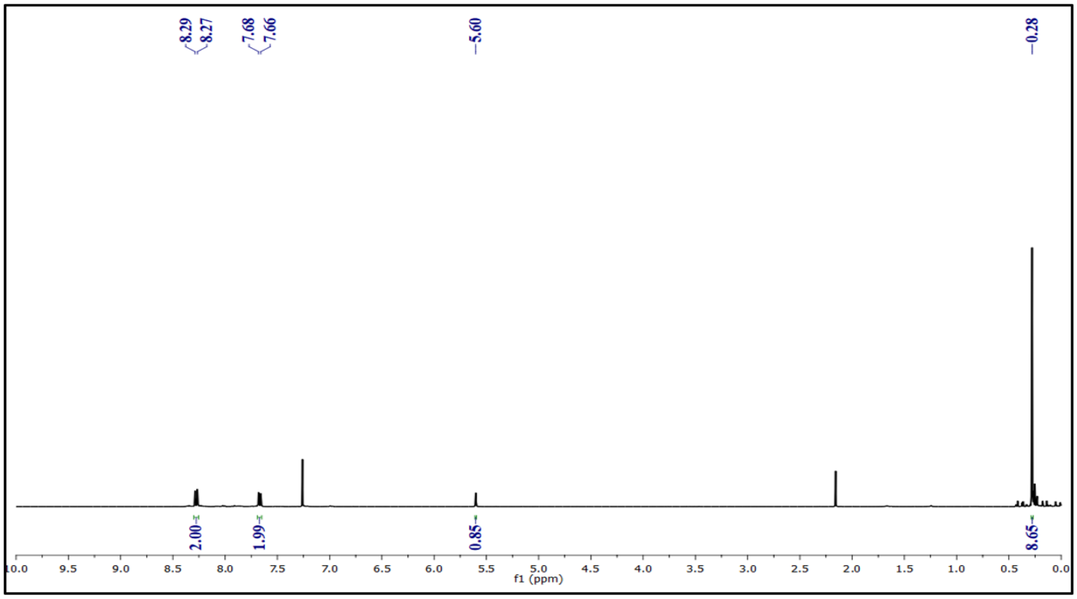

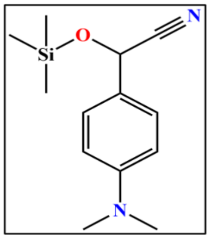

2-(4-(dimethylamino)phenyl)-2-((trimethylsilyl)oxy)acetonitrile:
^{1}H-NMR (400 MHz, $CDCl_3$, 25°C, TMS), δ (in ppm): 7.33 (d, J= 8 Hz, 2H, ArH), 6.72 (d, J= 8 Hz, 2H, ArH), 5.4 (s, 1H, ArH), 2.98 (s, 6H, Alkyl H), 0.21 (s, 9H, Alkyl H)

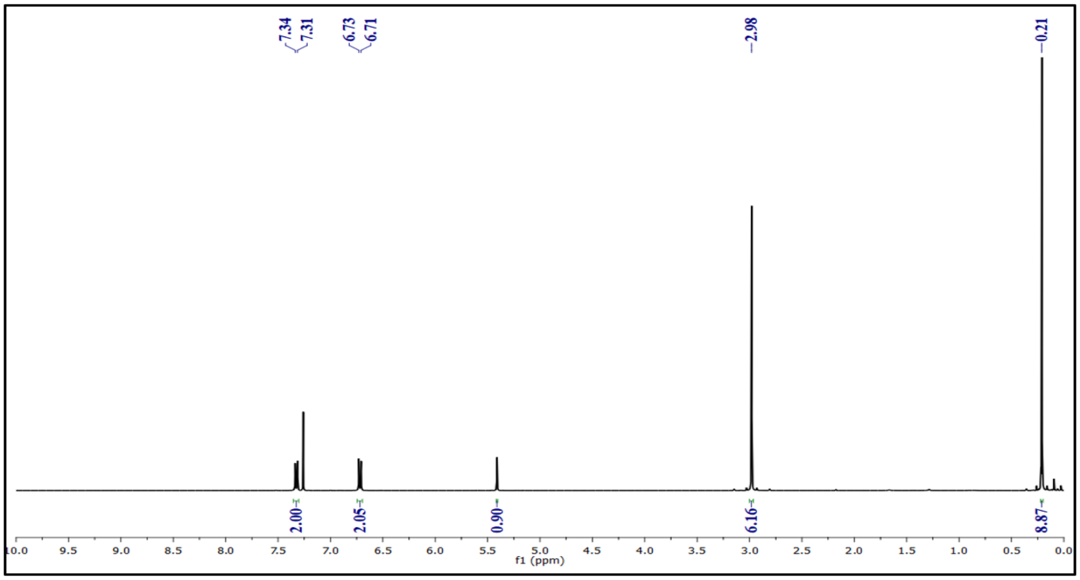

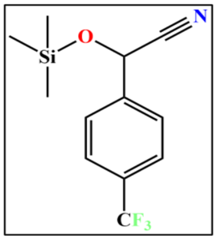

2-(4-(trifluoromethyl)phenyl)-2-((trimethylsilyl)oxy)acetonitrile:
^{1}H-NMR (400 MHz, $CDCl_3$, 25°C, TMS), δ (in ppm): 7.69 (d, J= 8 Hz, 2H, ArH), 7.61 (d, J= 8 Hz, 2H, ArH), 5.56 (s, 1H, ArH), 0.27 (s, 9H, Alkyl H)

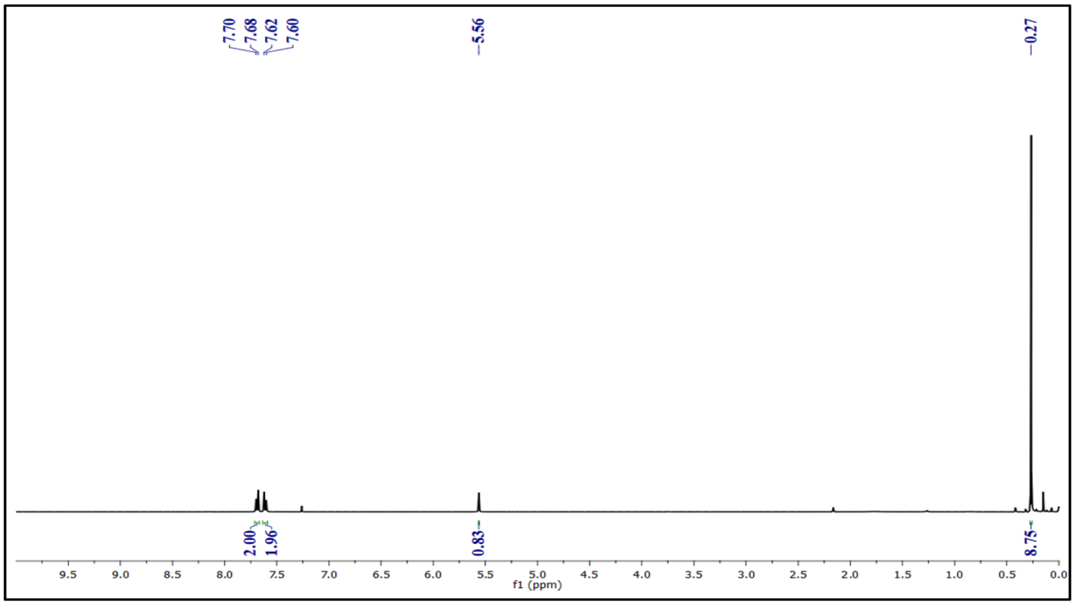

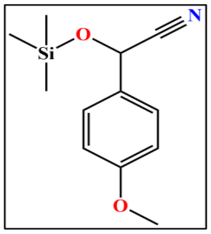

2-(4-methoxyphenyl)-2-((trimethylsilyl)oxy)acetonitrile:
^{1}H-NMR (400 MHz, $CDCl_3$, 25°C, TMS), δ (in ppm): 7.39 (d, J= 8 Hz, 2H, ArH), 6.93 (d, J= 8 Hz, 2H, ArH), 5.44 (s, 1H, ArH), 3.81 (s, 3H, Alkyl H), 0.21 (s, 9H, Alkyl H)

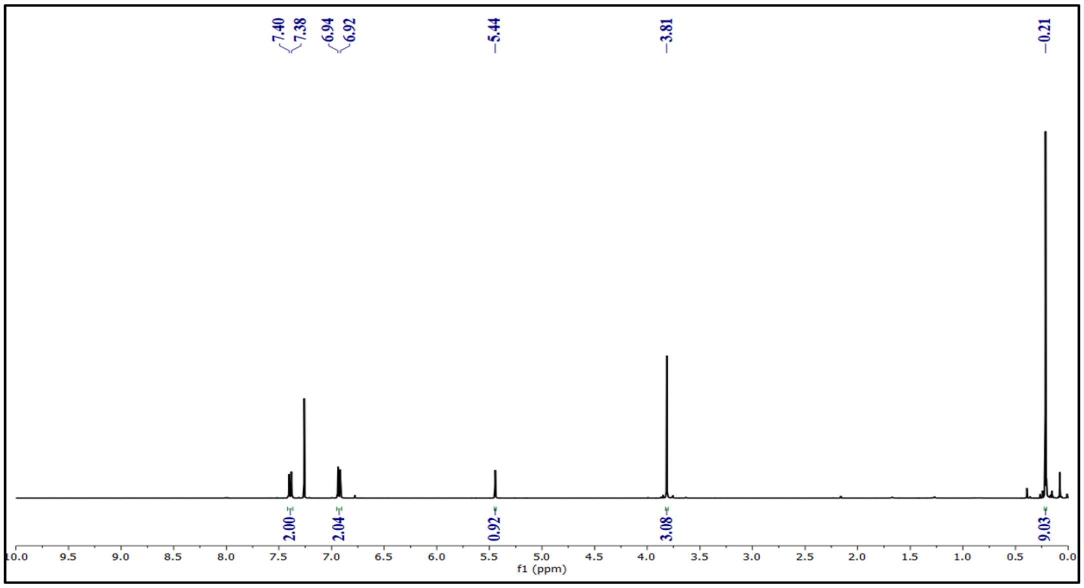

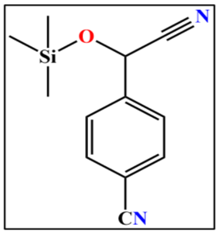

4-(cyano((trimethylsilyl)oxy)methyl)benzonitrile:
^{1}H-NMR (400 MHz, $CDCl_3$, 25°C, TMS), δ (in ppm): 7.71 (d, J= 8 Hz, 2H, ArH), 7.60 (d, J= 8 Hz, 2H, ArH), 5.55 (s, 1H, ArH), 0.26 (s, 9H, Alkyl H)

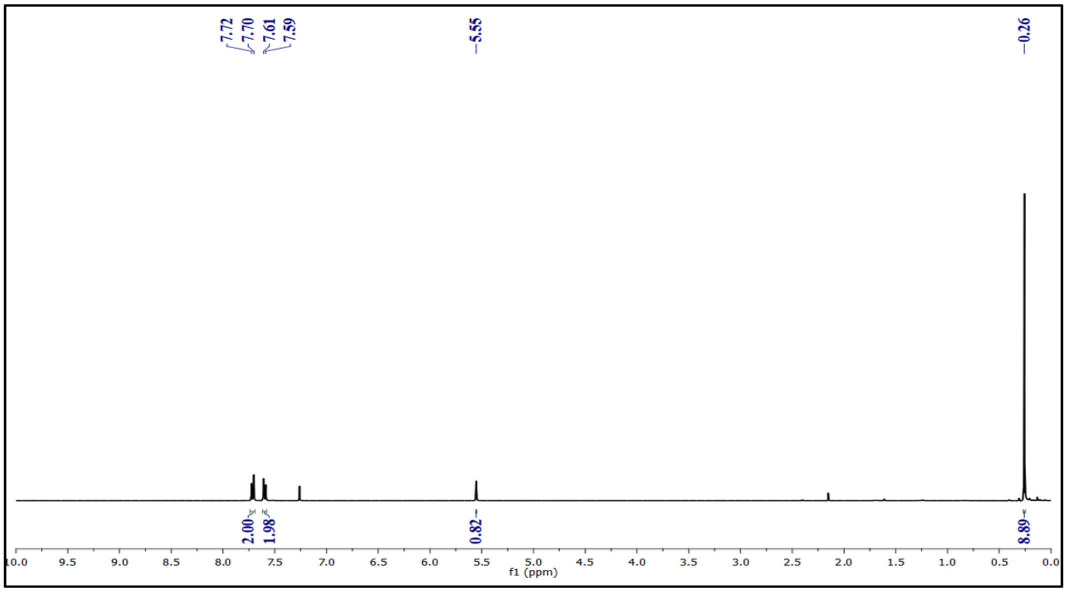

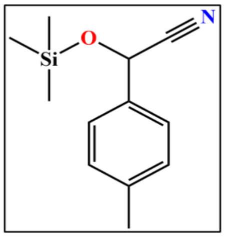

2-(p-tolyl)-2-((trimethylsilyl)oxy)acetonitrile:
^{1}H-NMR (400 MHz, $CDCl_3$, 25°C, TMS), δ (in ppm): 7.36 (d, 2H, ArH), 7.22 (d, J= 8 Hz, 2H, ArH), 5.47 (s, 1H, ArH), 2.28 (s, 3H, Alkyl H), 0.23 (s, 9H, Alkyl H)

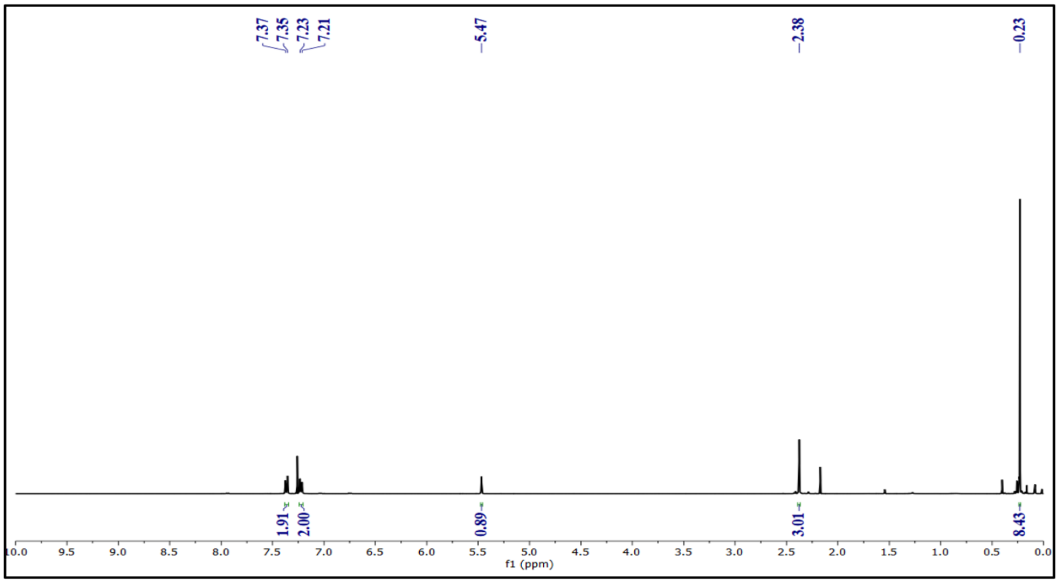

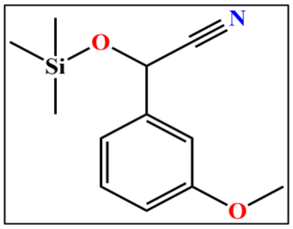

2-(3-methoxyphenyl)-2-((trimethylsilyl)oxy)acetonitrile:
^{1}H-NMR (400 MHz, $CDCl_3$, 25°C, TMS), δ (in ppm): 7.32 (t, J= 8 Hz, 1H, ArH), 7.04 (d, J= 8 Hz, 2H, ArH), 6.91 (d, J= 8 Hz, 1H, ArH), 5.47 (s, 1H, ArH), 3.05 (s, 3H, Alkyl H), 0.24 (s, 9H, Alkyl H)

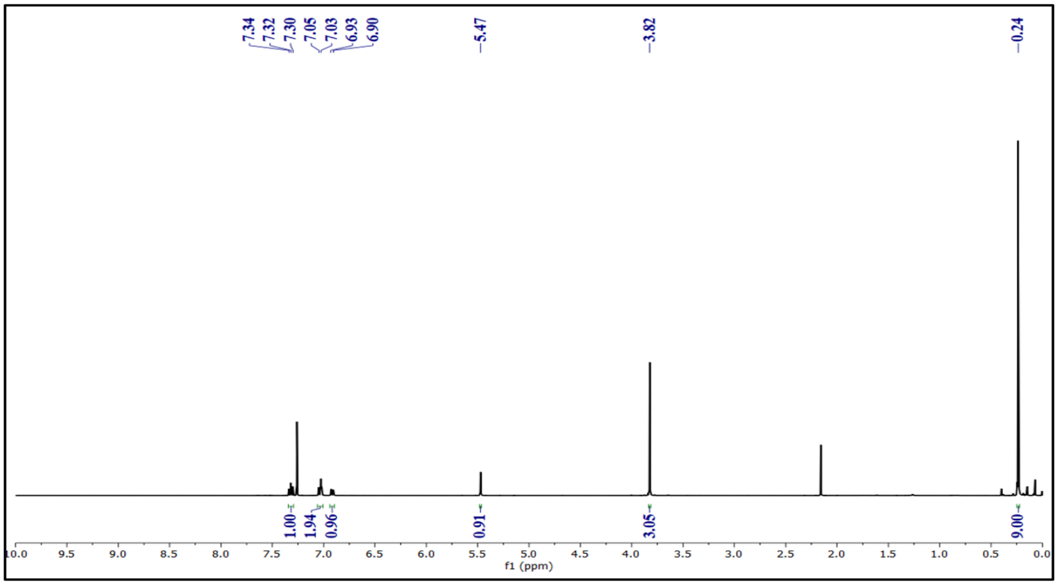

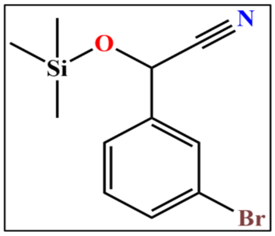

2-(3-bromophenyl)-2-((trimethylsilyl)oxy)acetonitrile:
^{1}H-NMR (400 MHz, $CDCl_3$, 25°C, TMS), δ (in ppm): 7.62 (t, J= 8 Hz, 1H, ArH), 7.51 (t, J= 8 Hz, 1H, ArH), 7.40 (t, J= 8 Hz, 1H, ArH), 7.31 (d, J= 8 Hz, 1H, ArH), 5.46 (s, 1H, ArH), 0.25 (s, 9H, Alkyl H)

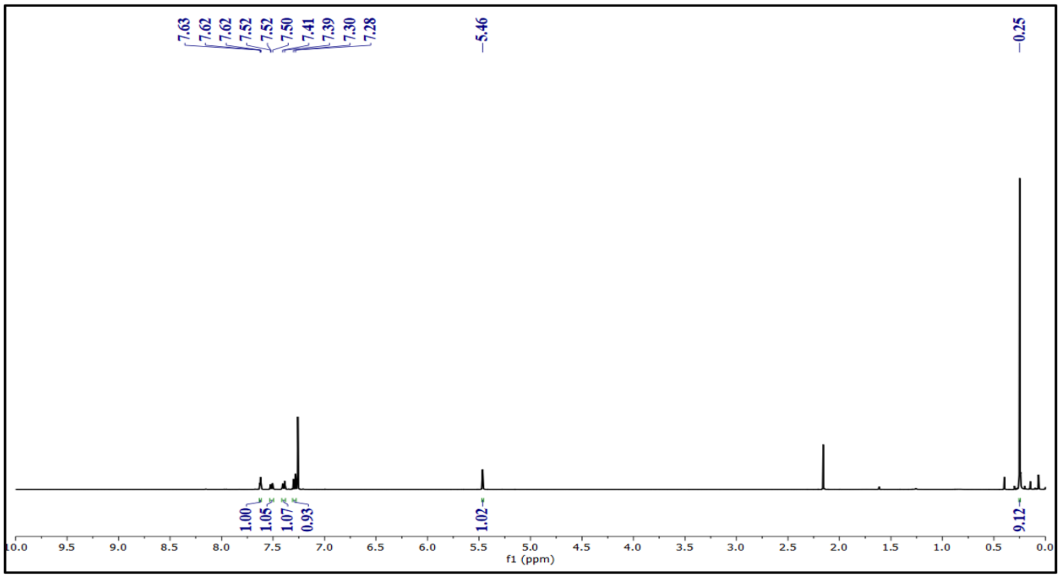

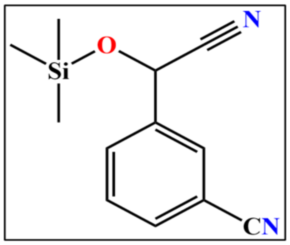

3-(cyano((trimethylsilyl)oxy)methyl)benzonitrile:
^{1}H-NMR (400 MHz, $CDCl_3$, 25°C, TMS), δ (in ppm): 7.77 (s, J= 8 Hz, 1H, ArH), 7.71 (d, J= 8 Hz, 1H, ArH), 7.67 (d, J= 8 Hz, 1H, ArH), 7.54 (d, J= 8 Hz, 1H, ArH), 5.54 (s, 1H, ArH), 0.26 (s, 9H, Alkyl H)

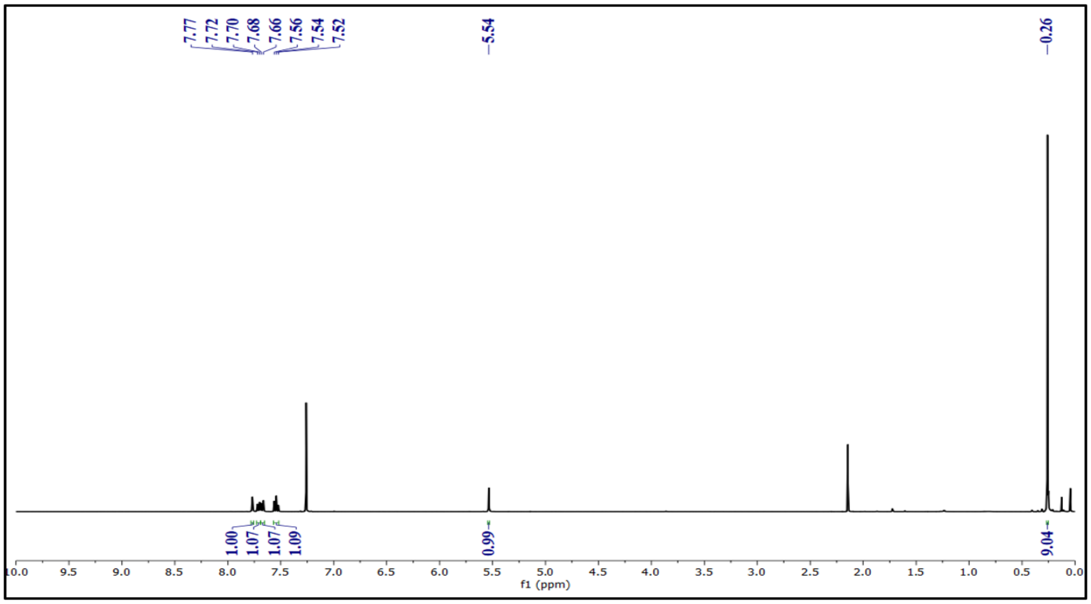

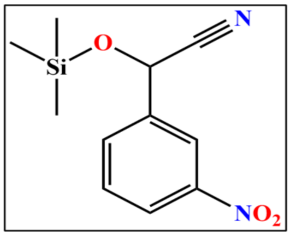

2-(3-nitrophenyl)-2-((trimethylsilyl)oxy)acetonitrile:
^{1}H-NMR (400 MHz, $CDCl_3$, 25°C, TMS), δ (in ppm): 8.34 (s, 1H, ArH), 8.25 (d, J= 8 Hz, 1H, ArH), 7.83 (d, J= 8 Hz, 1H, ArH), 7.63 (t, J= 8 Hz, 1H, ArH), 5.61 (s, 1H, ArH), 0.28 (s, 9H, Alkyl H)

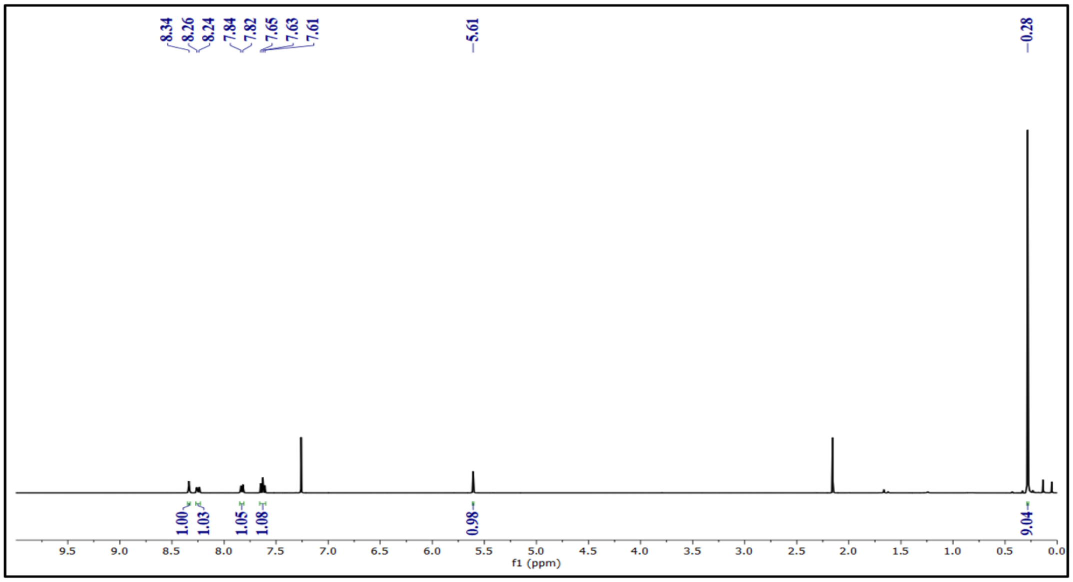

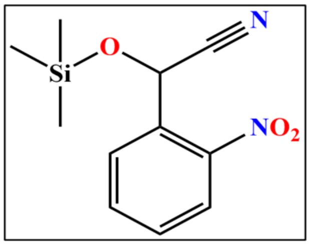

2-(2-nitrophenyl)-2-((trimethylsilyl)oxy)acetonitrile :
^{1}H-NMR (400 MHz, $CDCl_3$, 25°C, TMS), δ (in ppm): 8.16 (d, 8 Hz, 1H, ArH), 8.01 (t, J= 8 Hz, 1H, ArH), 7.77 (t, J= 8 Hz, 1H, ArH), 7.60 (m, J= 8 Hz, 1H, ArH), 6.20 (d, 1H, ArH), 0.28 (s, 9H, Alkyl H)

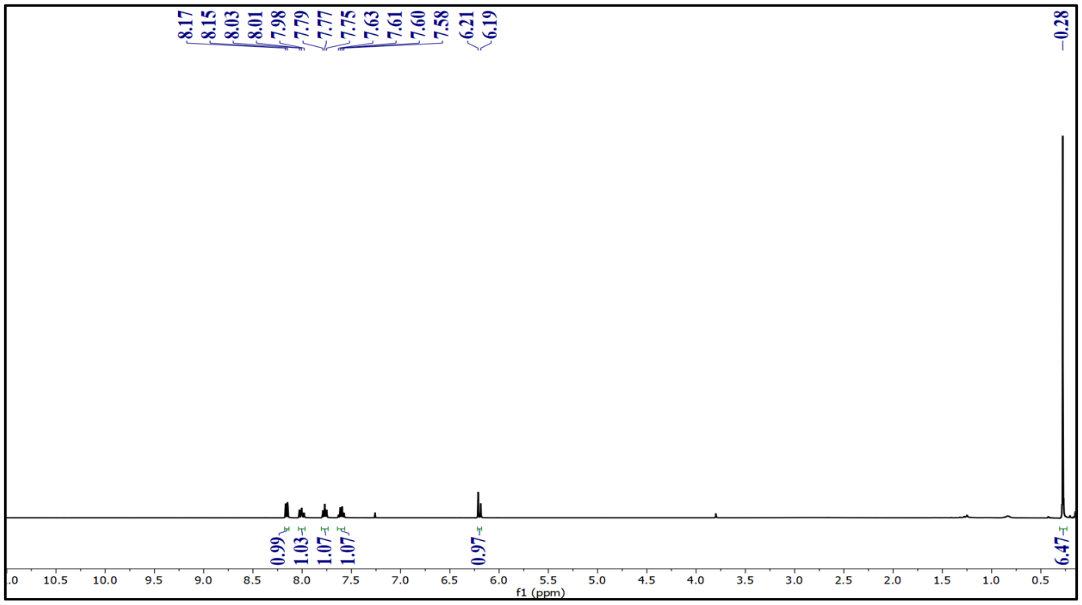

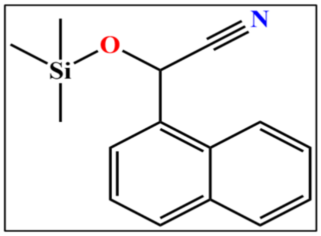

2-(naphthalen-1-yl)-2-((trimethylsilyl)oxy)acetonitrile :
^{1}H-NMR (400 MHz, $CDCl_3$, 25°C, TMS), δ (in ppm): 8.20 (d, 8 Hz, 1H, ArH), 7.91 (d, J= 8 Hz, 2H, ArH), 7.72 (d, J= 8 Hz, 1H, ArH), 7.63 (t, J= 8 Hz, 1H, ArH), 7.56 (t, J= 8 Hz, 1H, ArH), 7.49 (t, J= 8 Hz, 1H, ArH), 6.07 (s, 1H, ArH), 0.22 (s, 9H, Alkyl H)

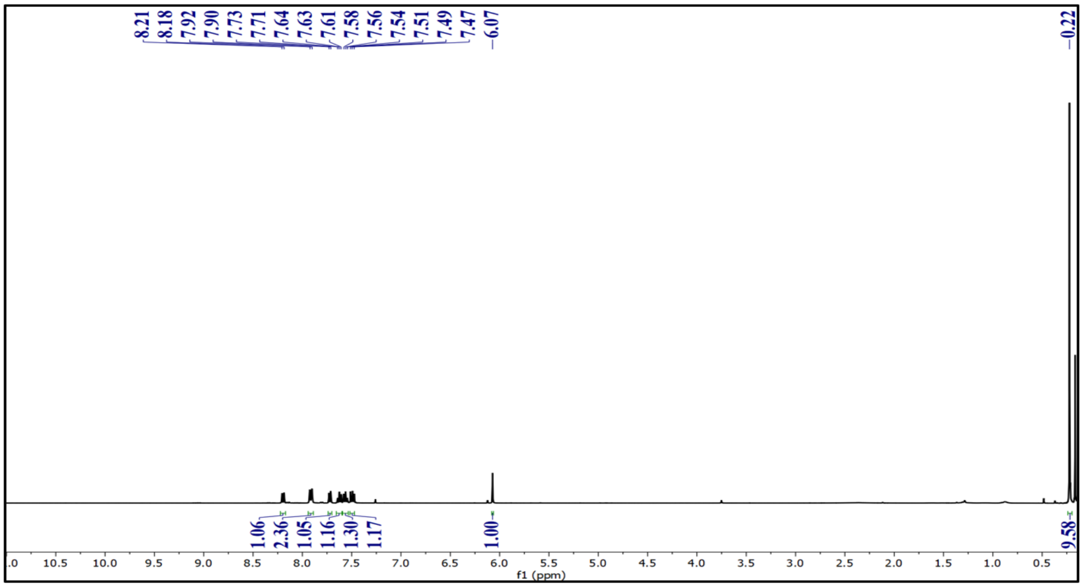

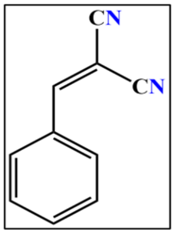

2-benzylidenemalononitrile:
^{1}H-NMR (400 MHz, $CDCl_3$, 25°C, TMS), δ (in ppm): 7.91 (d, J= 8 Hz, 2H, ArH), 7.78 (s, 1H, ArH), 7.64 (t, J= 8 Hz, 1H, ArH), 7.55 (t, 2H, J= 8 Hz, ArH)

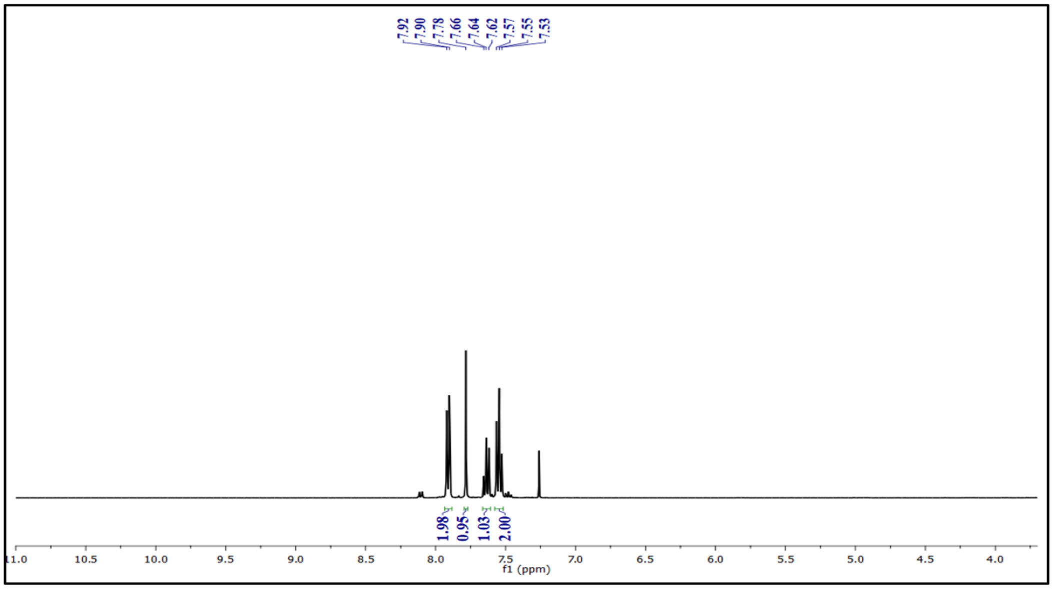

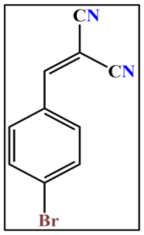

2-(4-bromobenzylidene)malononitrile:
^{1}H-NMR (400 MHz, $CDCl_3$, 25°C, TMS), δ (in ppm): 7.77 (d, J= 8 Hz, 2H, ArH), 7.72 (s, 1H, ArH), 7.69 (d, 2H, J= 8 Hz, ArH)

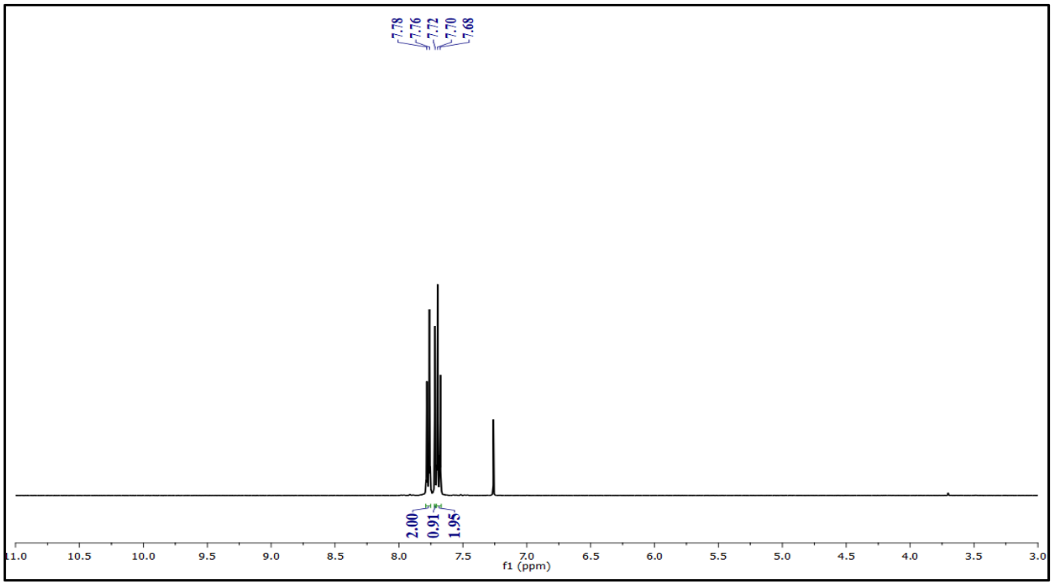

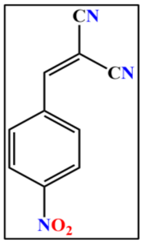

2-(4-nitrobenzylidene)malononitrile:
^{1}H-NMR (400 MHz, $CDCl_3$, 25°C, TMS), δ (in ppm): 8.39 (d, J= 8 Hz, 2H, ArH), 8.07 (d, 2H, J= 8 Hz, ArH), 7.89 (s, 1H, ArH)

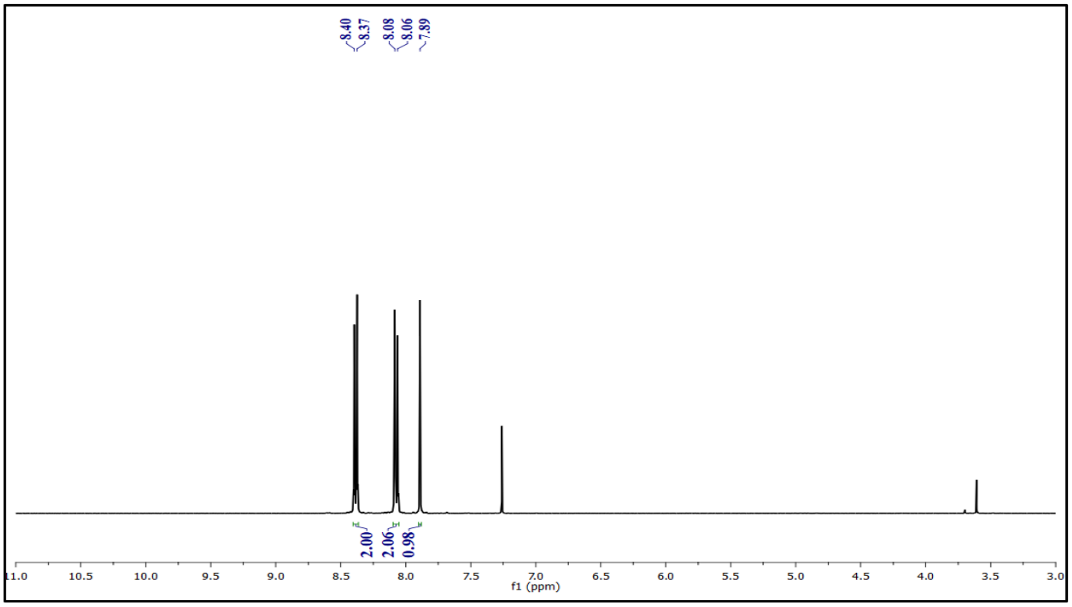

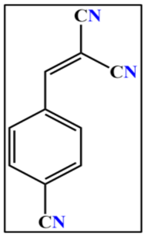

2-(4-cyanobenzylidene)malononitrile:
^{1}H-NMR (400 MHz, $CDCl_3$, 25°C, TMS), δ (in ppm): 8.00 (d, J= 8 Hz, 2H, ArH), 7.83 (d, 2H, J= 8 Hz, ArH), 7.82 (s, 1H, ArH)

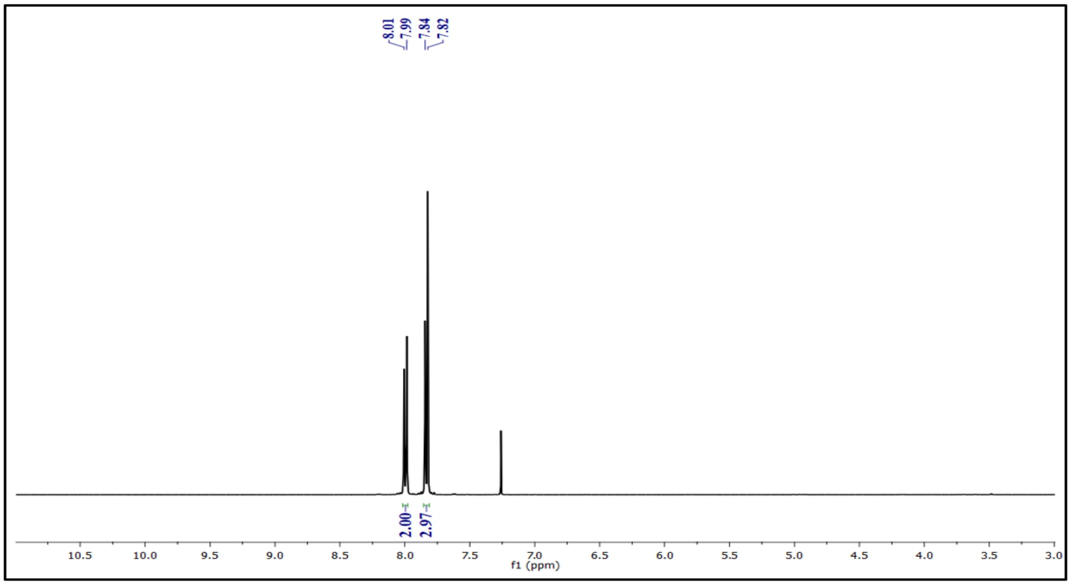

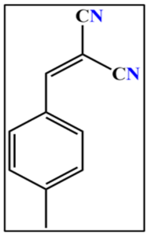

2-(4-methylbenzylidene)malononitrile:
^{1}H-NMR (400 MHz, $CDCl_3$, 25°C, TMS), δ (in ppm): 7.81 (d, J= 8 Hz, 2H, ArH), 7.72 (s, 1H, ArH), 7.34 (d, 2H, J = 8 Hz, ArH), 2.49 (s, 3H, Alkyl H)

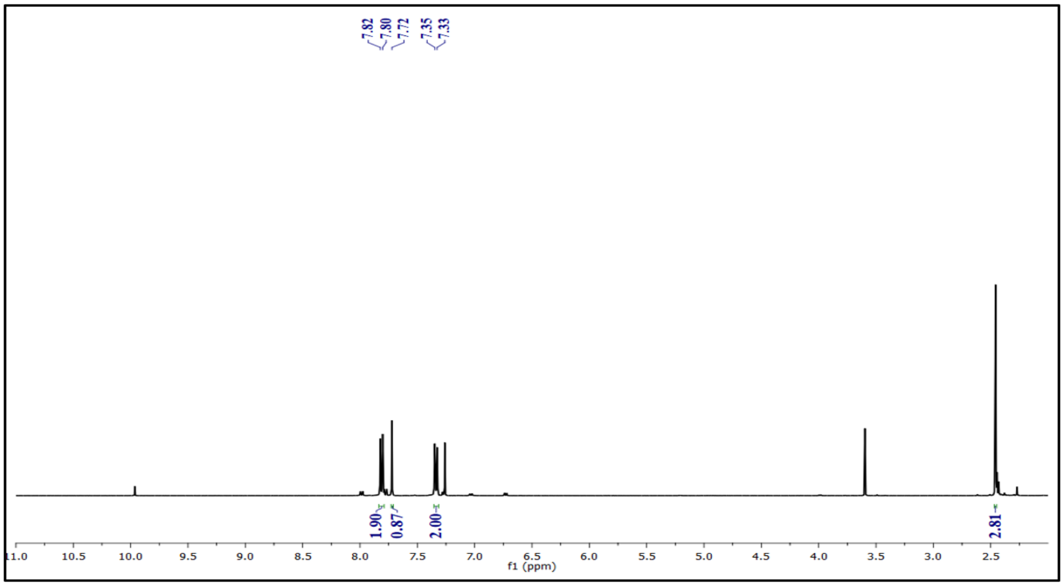

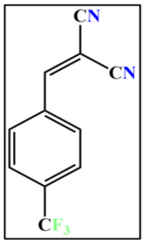

2-(4-(trifluoromethyl)benzylidene)malononitrile:
^{1}H-NMR (400 MHz, $CDCl_3$, 25°C, TMS), δ (in ppm): 8.02 (d, J= 8 Hz, 2H, ArH), 7.84 (s, 1H, ArH), 7.81 (d, 2H, J = 8 Hz, ArH)

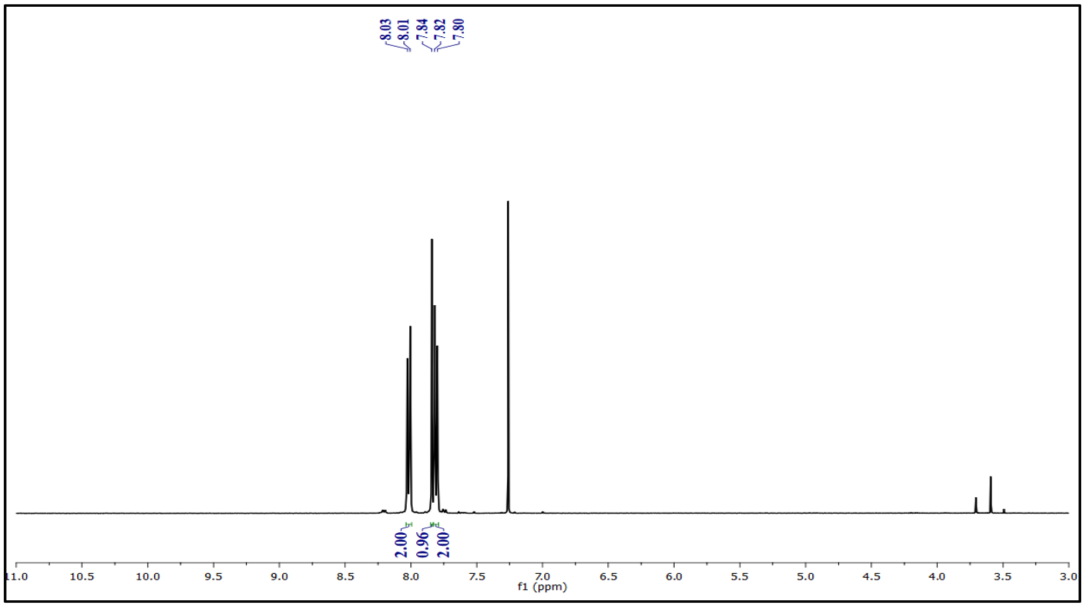

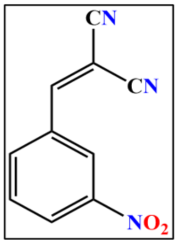

2-(3-nitrobenzylidene)malononitrile:
^{1}H-NMR (400 MHz, $CDCl_3$, 25°C, TMS), δ (in ppm): 8.67 (s, 1H, ArH), 8.47 (d, 1H, J= 8 Hz ArH), 8.31 (d, 1H, J= 8 Hz, ArH), 7.92 (s, 1H, ArH), 7.79 (t, 1H, J= 8 Hz, ArH)

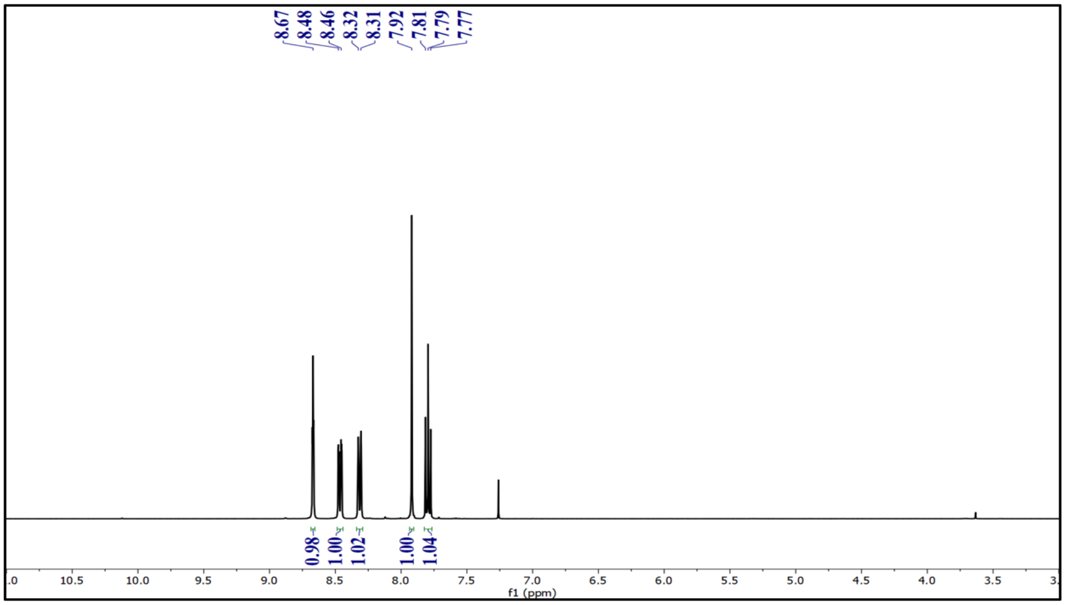

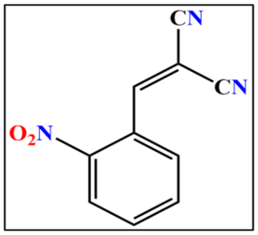

2-(2-nitrobenzylidene)malononitrile:
^{1}H-NMR (400 MHz, $CDCl_3$, 25°C, TMS), δ (in ppm): 8.45 (s, 1H, ArH), 8.35 (d, 1H, J= 8 Hz ArH), 7.88 (t, 1H, J= 8 Hz, ArH), 7.81 (t, 2H, J= 8 Hz, ArH)

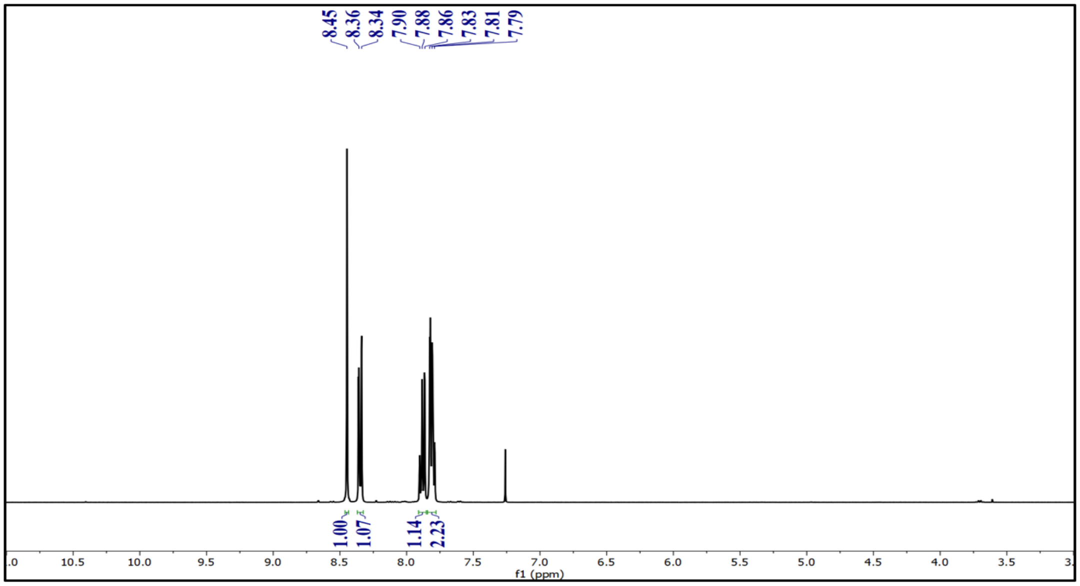

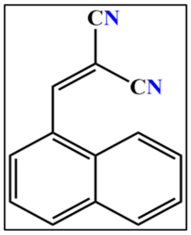

2-(naphthalen-1-ylmethylene)malononitrile:
^{1}H-NMR (400 MHz, $CDCl_3$, 25°C, TMS), δ (in ppm): 8.64 (s, 1H, ArH), 8.27 (d, 1H, J= 8 Hz ArH), 8.10 (d, 1H, J= 8 Hz, ArH), 7.95 (d, 2H, J= 8 Hz, ArH), 7.64 (m, 3H, J= 8 Hz, ArH)

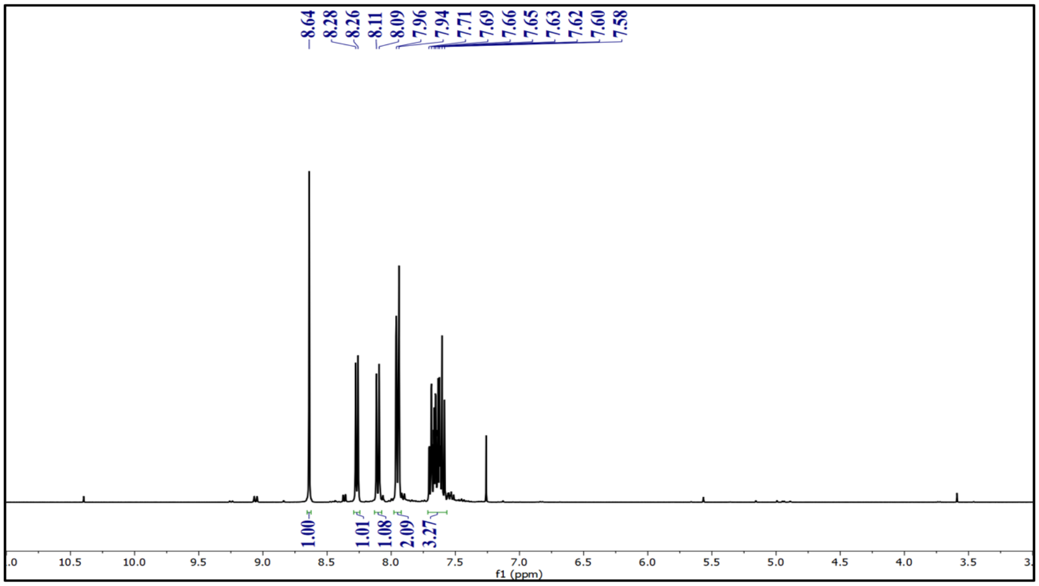

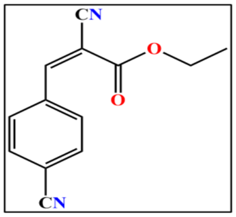

Ethyl-2-cyano-3-(4-cyanophenyl)acrylate:
^{1}H-NMR (400 MHz, $CDCl_3$, 25°C, TMS), δ (in ppm): 8.24 (s, 1H, ArH), 8.06 (d, J= 8 Hz, 2H, ArH), 7.79 (d, J= 8 Hz, 2H, ArH), 4.41 (m, J= 8 Hz, 2H), 1.41 (t, J= 8 Hz, 3H)

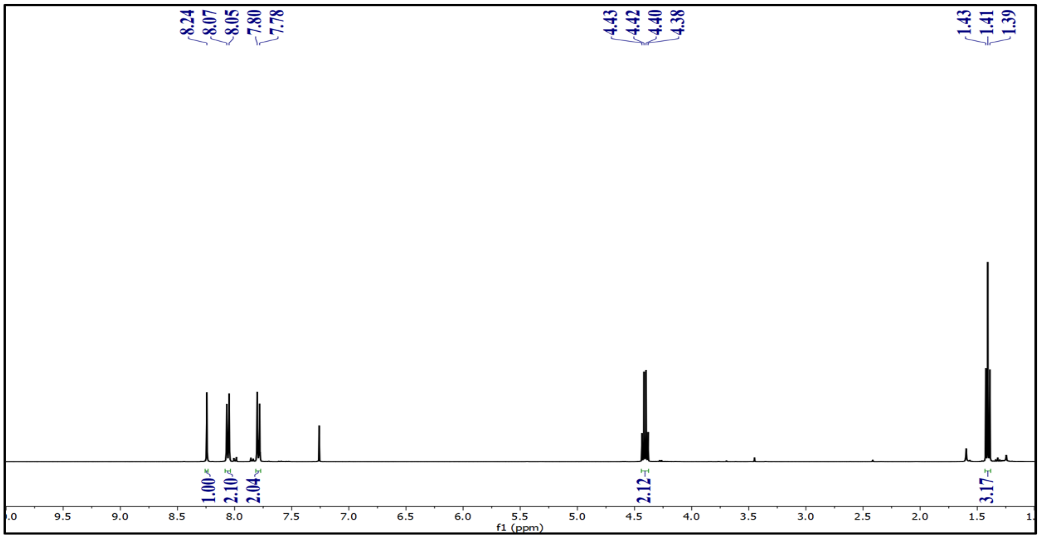

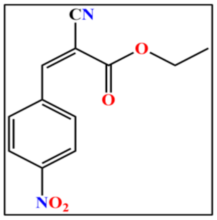

Ethyl-2-cyano-3-(4-nitrophenyl)acrylate:
^{1}H-NMR (400 MHz, $CDCl_3$, 25°C, TMS), δ (in ppm): 8.35 (d, J= 8 Hz, 2H, ArH), 8.30 (s, 1H, ArH), 8.13 (d, J= 8 Hz, 2H, ArH), 4.42 (m, J= 8 Hz, 2H), 1.42 (t, J= 8, Hz 3H)

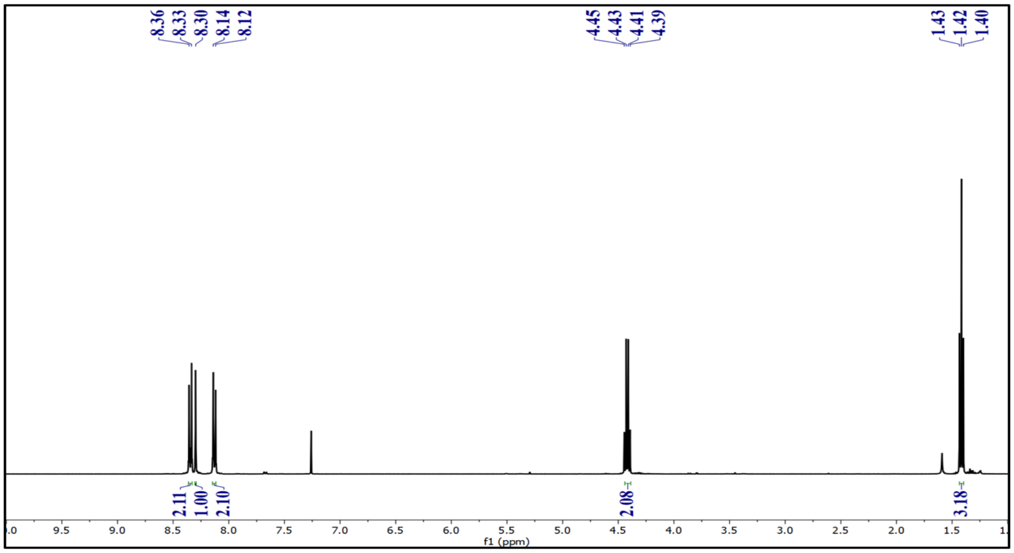

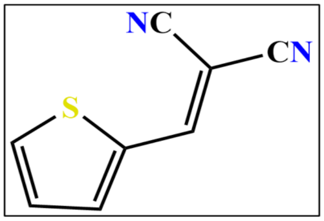

2-(thiophen-2-ylmethylene)malononitrile:
^{1}H-NMR (400 MHz, $CDCl_3$, 25°C, TMS), δ (in ppm): 7.89 (s, 1H, ArH), 7.87 (s, 1H, ArH), 7.81 (d, J= 8 Hz, 1H, ArH), 7.26 (t,1H, ArH)

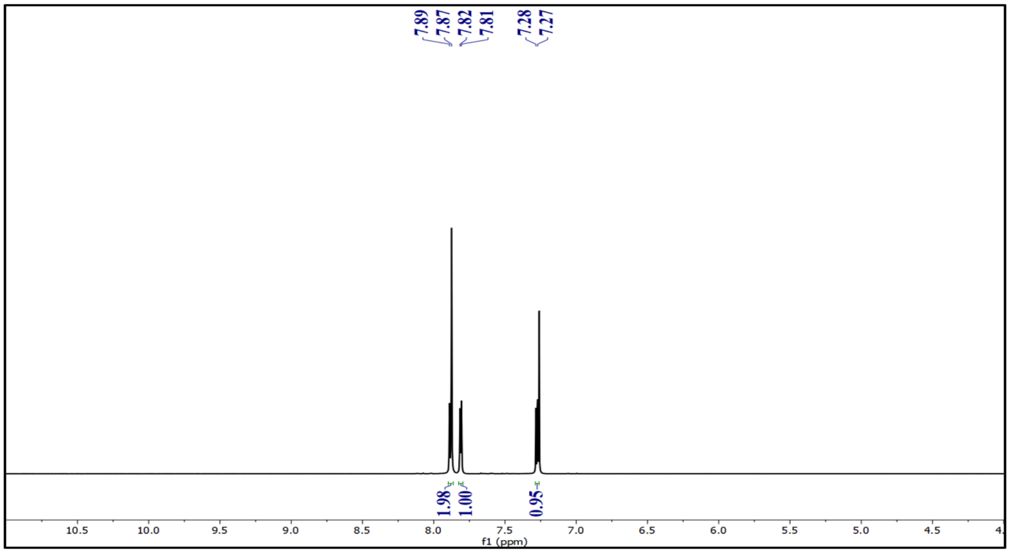

Section S6: ^{1}H NMR spectra of continuous flow cascade catalysis

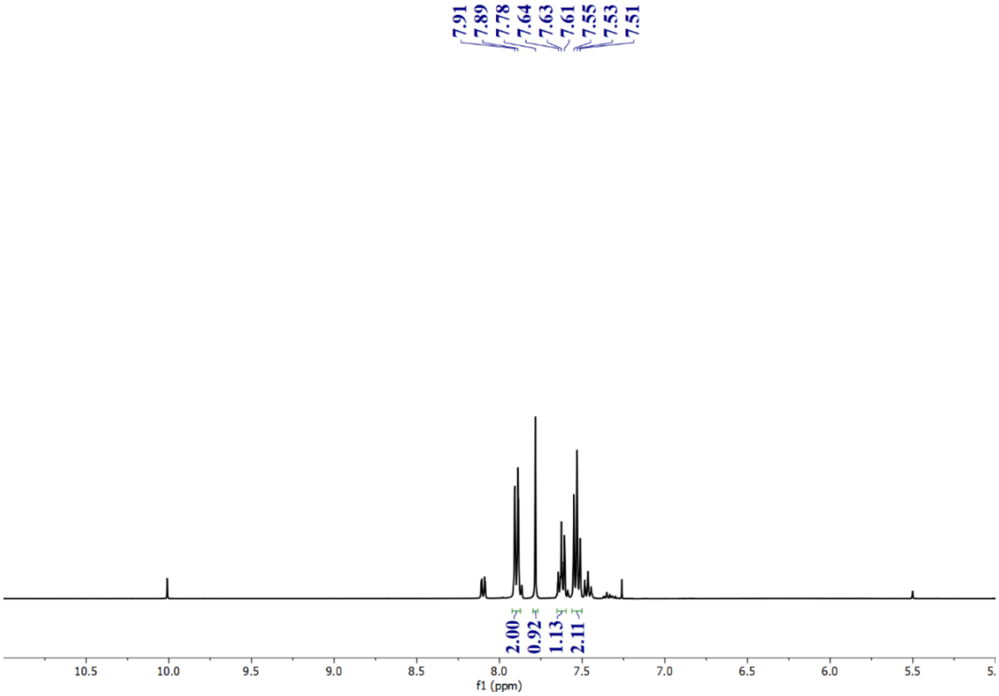

Figure S21: ^{1}H NMR spectra ($CDCl_3$) of the cascade catalysis under continuous flow (Run 1)

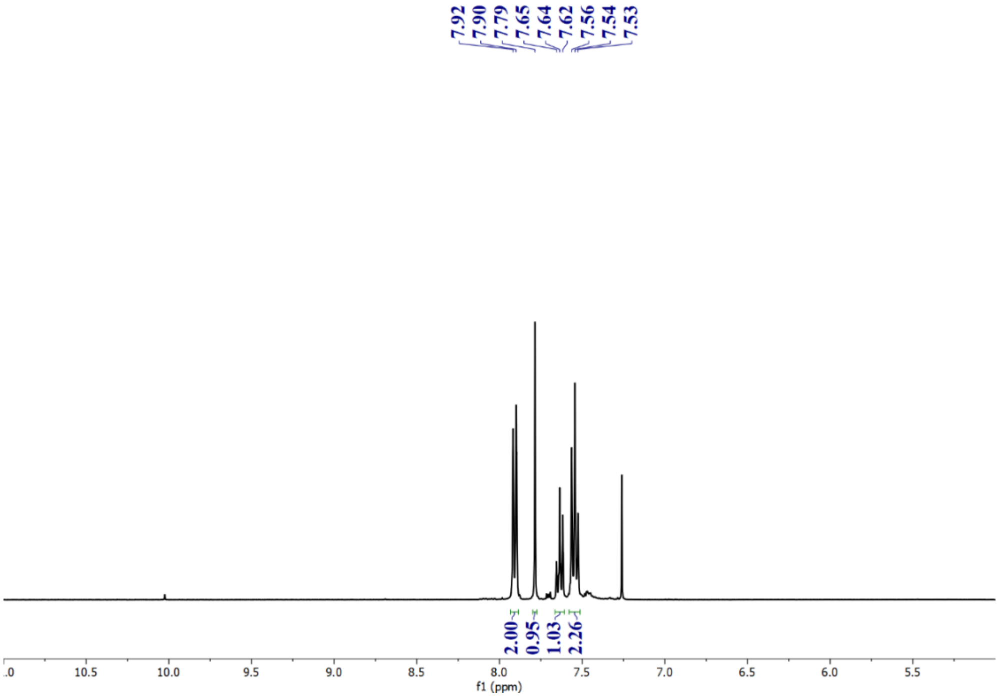

Figure S22: ^{1}H NMR spectra ($CDCl_3$) of the cascade catalysis under continuous flow (Run 2)

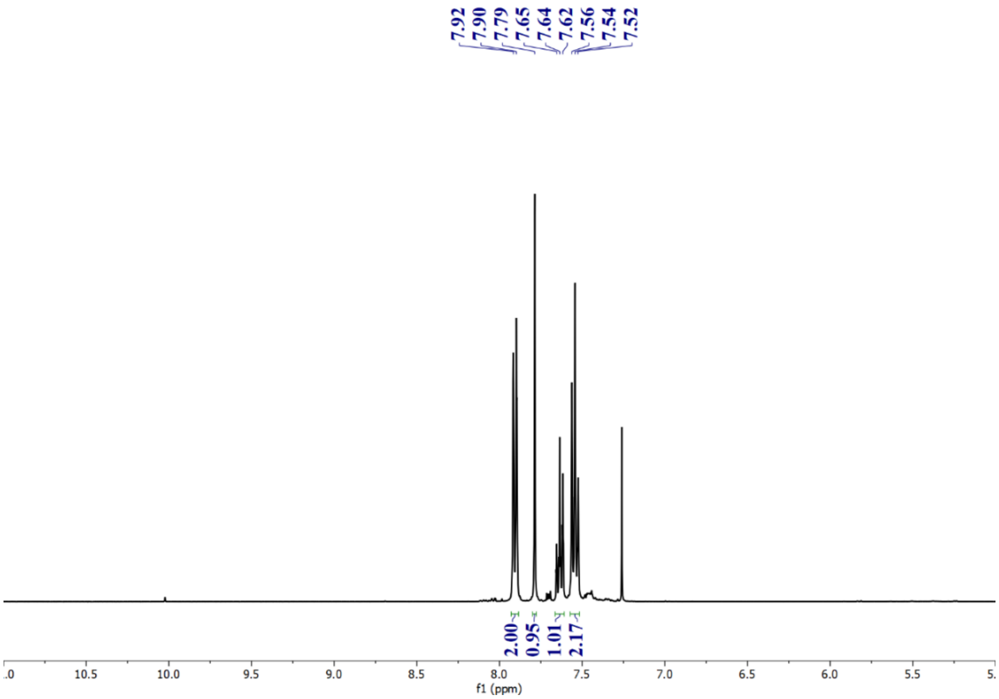

Figure S23: ^{1}H NMR spectra ($CDCl_3$) of the cascade catalysis under continuous flow (Run 3)

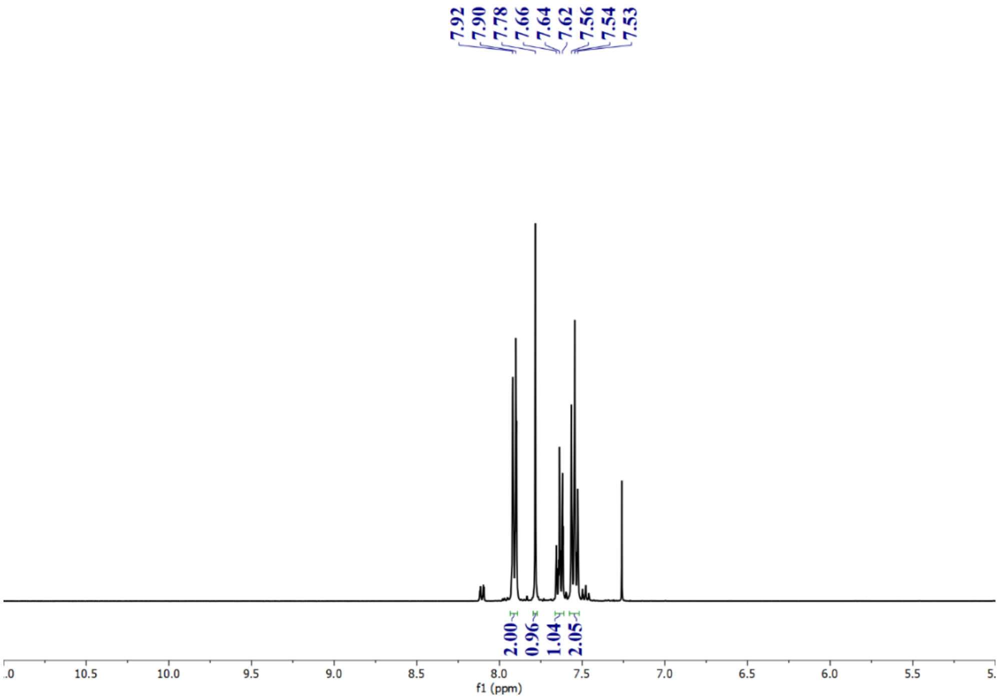

Figure S24: ^{1}H NMR spectra ($CDCl_3$) of the cascade catalysis under continuous flow (Run 4)

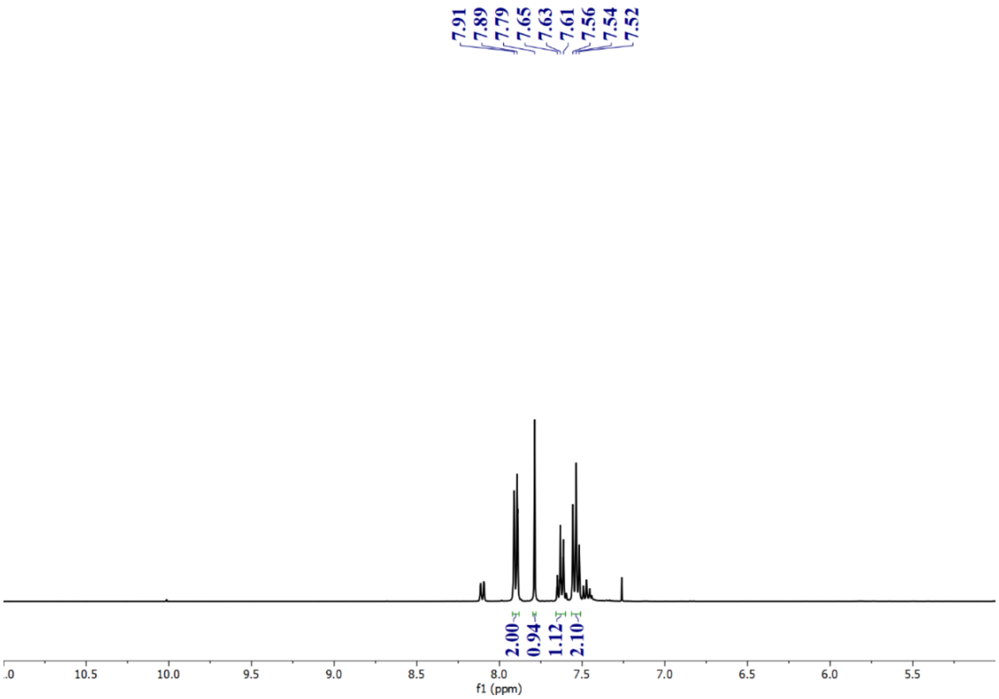

Figure S25: ^{1}H NMR spectra ($CDCl_3$) of the cascade catalysis under continuous flow (Run 5)

Table S4. Substrate scope for 120-MI@OH catalyzed one-pot cascade reaction

Entry	Substrate	Product	Yield
1	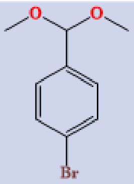	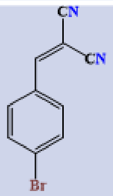	>99 %
2	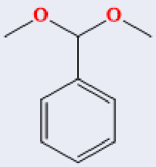	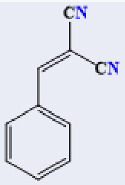	>99 %

Table S5. One-pot cascade catalysis activity, parameters comparison of 120-MI@OH with other previously reported organic polymers

Type	Catalyst	Reaction condition (Temp./ Solvent)	Duration	Yield (%)	Reusability (Cycles)	Flow catalysis	Reference
Polymer/ COF	**120-MI@OH**	**60 °C, H_2O**	**2 h**	**> 99**	**5**	**Yes**	**This Work**
	HisAA-220	80 °C, MeCN	4 h	77	5	No	2
	3D DL-COF-1	RT, $CDCl_3$	20 h	98	3	No	3
	2,3-DhaTph	80 °C, Toluene	1 h	96	5	No	4
	PPAF-SO_3H + PPAF-NH_2	90 °C, Toluene	1 h	87	7	No	5
	TAPB-DMTP-PA-COF	75°C, Solvent-free	5 h	99	15	Yes	6
	PPAF-SO_3H-NH_2	90 °C, Toluene	1 h	100	8	No	7
	HCPs–SO_3H/ CH_2NH_2	90 °C, Toluene	7 h	95	3	No	8
	PPVBS/HPONNs + PVBMAP/HPONNs	80 °C, Toluene	2 h	98	12	No	9
	P(DVB–NH_2–0.5-StSO_3H)	80 °C, Toluene	24 h	99	5	No	10
	CBAP-1(EDA-SO_3H)	70 °C, Toluene	2h	100	7	No	11

References:

1. S. Let, S. Dutta, P. Samanta, S. Sharma and S. K. Ghosh, Magnetic nanoparticle-embedded ionic microporous polymer composite as an efficient scavenger of organic micropollutants. *ACS Appl. Mater. Interfaces* **2021**, 13, 51474−51484.

2. S. Rat, A. Chavez-Sanchez, M. Jerigová, D. Cruz, M. Antonietti, Acetic anhydride polymerization as a pathway to functional porous organic polymers and their application in acid–base catalysis, *ACS Appl. Polym. Mater.*, **2021**, 3, 2588-2597.

3. H. Li, Q. Pan, Y. Ma, X. Guan, M. Xue, Q. Fang, Y. Yan, V. Valtchev, S. Qiu, Three-dimensional covalent organic frameworks with dual linkages for bifunctional cascade catalysis, *J. Am. Chem. Soc.*, **2016**, 138, 14783-14788.

4. D. B. Shinde, S. Kandambeth, P. Pachfule, R. R. Kumar, R. Banerjee, Bifunctional covalent organic frameworks with two dimensional organocatalytic micropores, *Chem. Commun.*, **2015**, 51, 310- 313.

5. E. Merino, E. Verde-Sesto, E. M. Maya, A. Corma, M. Iglesias, F. Sánchez, Mono-functionalization of porous aromatic frameworks to use as compatible heterogeneous catalysts in one-pot cascade reactions, *Appl. Catal. A: Gen.*, **2014**, 469, 206-212.

6. X.-T. Li, J. Zou, Q. Yu, Y. Liu, J.-R. Li, M.-J. Li, H.-C. Ma, G.-J. Chen and Y.-B. Dong, Construction of acid–base bifunctional covalent organic frameworks via Doebner reaction for catalysing cascade reaction, *Chem. Commun.*, **2022**, 58, 2508–2511.

7. E. Merino, E. Verde-Sesto, E. M. Maya, M. Iglesias, F. Sánchez, A. Corma, Synthesis of structured porous polymers with acid and basic sites and their catalytic application in cascade-type reactions, *Chem. Mater.*, **2013**, 25, 981-988.

8. K. Wang, Z. Jia, X. Yang, L. Wang, Y. Gu and B. Tan, Acid and base coexisted heterogeneous catalysts supported on hypercrosslinked polymers for one-pot cascade reactions, *J. Catal.*, **2017**, 348, 168-176.

9. L. Zhang, H. Yu, S. Gao, H. Wang, Z. He and K. Huang, In Situ Synthesis of Incompatible Polymers within Hollow Porous Organic Nanosphere Networks for Cascade *Reactions, Macromol. Chem. Phys.*, **2021**, 222, 2100009.

10. Z. Sun, X. Yang, X. Huang, M. Zhang, G. Bian, Y. Qi, X. Yang and W. Zhang, Mesoporous polymeric catalysts with both sulfonic acid and basic amine groups for the one-pot deacetalization−Knoevenagel reaction, *New J. Chem.*, **2019** 43, 16676.

11. Puthiaraj,P.; Chung,Y.; Ahn,W. Dual-Functionalized Porous Organic Polymer as Reusable Catalyst for One-Pot Cascade C-C Bond-Forming Reactions, *Mol. Catal.*, **2017**,441,1−9.
